# Age-Associated Lipidome Changes in Metaphase II Mouse Oocytes

**DOI:** 10.1371/journal.pone.0148577

**Published:** 2016-02-16

**Authors:** Hyuck Jun Mok, Hyejin Shin, Jae Won Lee, Geun-Kyung Lee, Chang Suk Suh, Kwang Pyo Kim, Hyunjung Jade Lim

**Affiliations:** 1 Department of Applied Chemistry, The Institute of Natural Science, Kyung Hee University, Yongin, Gyeonggi-do, Korea; 2 Department of Biomedical Science & Technology, Institute of Biomedical Science & Technology, Konkuk University, Seoul, Korea; 3 Department of Obstetrics and Gynecology, Seoul National University Bundang Hospital, Seongnam, Gyeonggi-do, Korea; 4 Department of Veterinary Medicine, Konkuk University, Seoul, Korea; Max-Delbrück Center for Molecular Medicine (MDC), GERMANY

## Abstract

The quality of mammalian oocytes declines with age, which negatively affects fertilization and developmental potential. The aging process often accompanies damages to macromolecules such as proteins, DNA, and lipids. To investigate if aged oocytes display an altered lipidome compared to young oocytes, we performed a global lipidomic analysis between oocytes from 4-week-old and 42 to 50-week-old mice. Increased oxidative stress is often considered as one of the main causes of cellular aging. Thus, we set up a group of 4-week-old oocytes treated with hydrogen peroxide (H_2_O_2_), a commonly used oxidative stressor, to compare if similar lipid species are altered between aged and oxidative-stressed oocytes. Between young and aged oocytes, we identified 26 decreased and 6 increased lipids in aged oocytes; and between young and H_2_O_2_-treated oocytes, we identified 35 decreased and 26 increased lipids in H_2_O_2_-treated oocytes. The decreased lipid species in these two comparisons were overlapped, whereas the increased lipid species were distinct. Multiple phospholipid classes, phosphatidic acid (PA), phosphatidylinositol (PI), phosphatidylserine (PS), and lysophosphatidylserine (LPS) significantly decreased both in H_2_O_2_-treated and aged oocytes, suggesting that the integrity of plasma membrane is similarly affected under these conditions. In contrast, a dramatic increase in diacylglycerol (DG) was only noted in H_2_O_2_-treated oocytes, indicating that the acute effect of H_2_O_2_-caused oxidative stress is distinct from aging-associated lipidome alteration. In H_2_O_2_-treated oocytes, the expression of lysophosphatidylcholine acyltransferase 1 increased along with increases in phosphatidylcholine. Overall, our data reveal that several classes of phospholipids are affected in aged oocytes, suggesting that the integrity of plasma membrane is associated with maintaining fertilization and developmental potential of mouse oocytes.

## Introduction

In most mammals, the fertilization and developmental competence of embryos are related to reproductive aging. Oocyte quality decreases with age, accompanied by several ultrastructural changes such as dilation of the smooth endoplasmic reticulum and Golgi complexes and higher frequency of ruptured mitochondrial membranes [1]. The oocytes from aged mice (hereafter referred to as aged oocytes) exhibit aging-related changes including aneuploidy, meiotic spindle abnormalities, and mitochondrial dysfunction [2–4].

Lipids exhibit tremendous structural variety with diverse head groups and fatty acid chains [5]. Understandably, with this diversity in types and structures, lipids are widely involved in a wide variety of biological functions. Their major roles include serving as constituents of cellular membranes in the form of lipid bilayers [6], energy storage in the form of chemical energy [7], and as precursors of secondary messengers that can be activated after cellular stimulation [8–11]. Lipids also play an important role in oocyte maturation and developmental competence [12–14]. Fatty acids stored as triacylglyceride (TG) within lipid droplets in oocytes and cumulus cells are oxidized by mitochondria via β-oxidation to provide energy sources during oocyte development [14]. Cumulus cells have been shown to protect maturing oocytes from fatty acid-induced lipotoxicity [15]. In humans, free fatty acid levels in follicular fluids are associated with poor morphology of cumulus-oocyte complexes (COCs) [13]. Poor developmental competence and low survival rates in pig oocytes after cryopreservation are also associated with high lipid content in lipid droplets [16]. Phospholipids, the major components of membranes, affect membrane fluidity and permeability, which are crucial parameters during post-cryopreservation survival of oocytes and embryos [17, 18]. In addition, it has been shown that phospholipids in the plasma membrane are vulnerable to oxidative stress [19–21].

Multiple causes of cellular aging have been suggested and the increased oxidative stress is one of them [22]. Ovarian aging is shown to accompany protein oxidation, DNA damage, and lipid peroxidation [23, 24]. Accumulation of oxidative stress in oocytes and surrounding ovarian cells of aged females eventually results in compromised oocyte viability and pregnancy rates [25]. A transcriptome analysis has also shown that several genes involved in managing oxidative stress or damage are downregulated in aged oocytes [26]. However, the widely accepted notion that the oxidative stress is an inducer of aging has somewhat lost its firm ground in recent years with the accumulating evidence which shows the opposite effect of oxidative stress in aging [27].

In lipidomics, many state-of-the-art technologies have been applied. In particular, one technique based on liquid chromatography (LC) coupled to electrospray ionization mass spectrometry (ESI/MS) is commonly employed for global quantitative analysis of lipid species in complex biological samples [5, 28–31]. LC is advantageous when the samples are of high complexity, because the chromatographic separation allows analyses of various lipid species [32–34]. However, peak tailing of acidic lipid classes including phosphatidic acid (PA), phosphatidylinositol (PI), phosphatidylserine (PS), lysophosphatidylserine (LPS), lysophosphatidic acid (LPA), lysophosphatidylinositol (LPI), and ceramide-1-phosphate (Cer1P) makes LC-based separation and quantitative analysis challenging. We applied a relatively rapid and easy derivatization method by adding a methyl group to the phosphate group to reduce its metal affinity to reduce peak tailing [28].

While it is generally recognized that oxidative stress is associated with ovarian aging [35, 36], whether overall lipidome is affected similarly in aged and oxidative-stressed oocytes is not known. In this study, we applied a novel lipidomics platform combined with this derivatization strategy for analysis of lipid alterations in exogenously oxidative-stressed and aged oocytes. We herein report the identification and quantification of various lipids that are differentially or similarly regulated under these conditions in oocytes.

## Results

### Visualization of intracellular lipids and plasma membrane in young and aged oocytes

**Fig 1:**
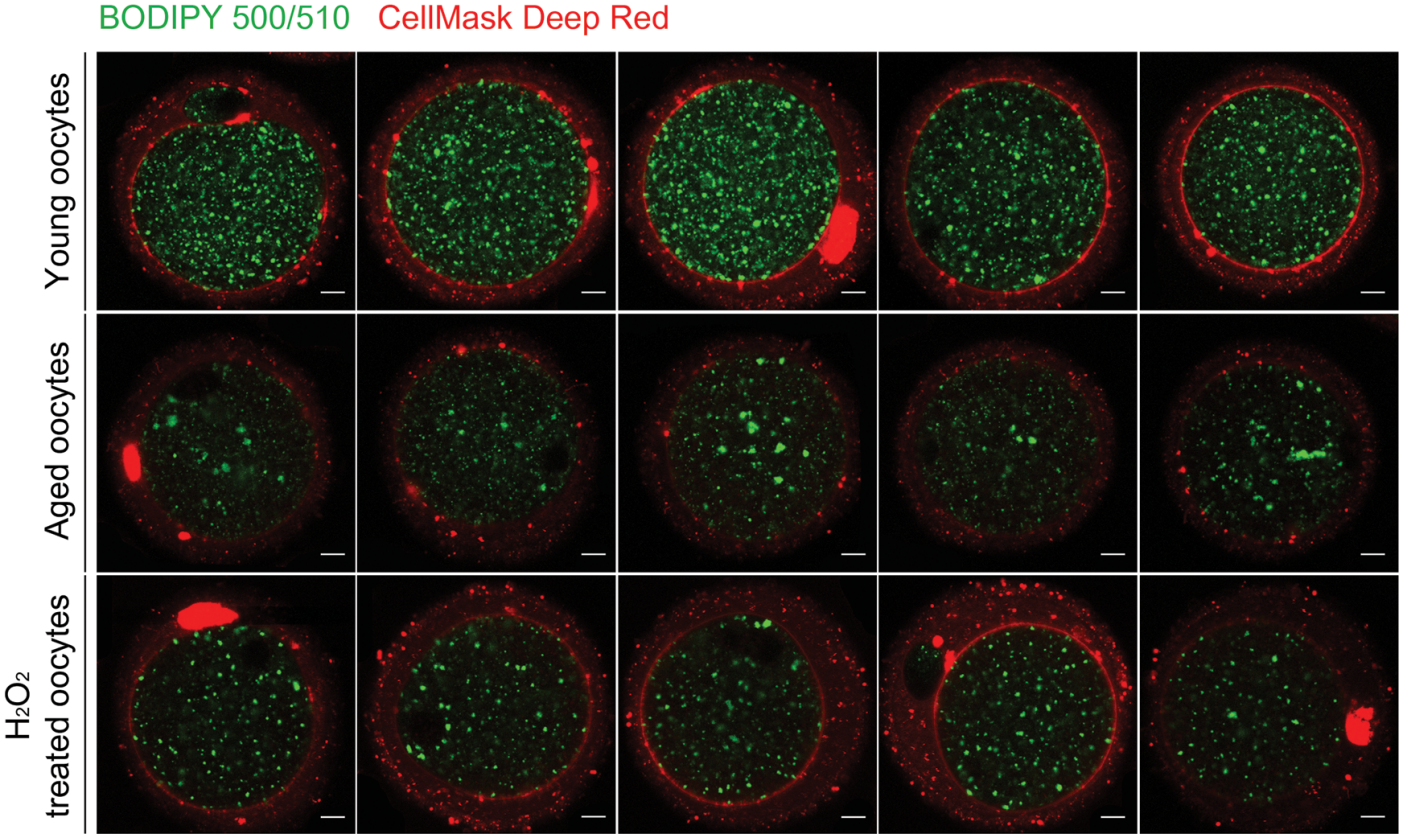
Fluorescence staining using BODIPY 500/510 and CellMask in young, aged, and H_2_O_2_-treated oocytes. Five oocytes from each group were stained with BODIPY 500/510 (10 mg/mL, green fluorescence) and CellMask Deep Red (2.5 mg/mL, red fluorescence) in PBS. Oocytes from 4-week-old mice were treated with 50 μM H_2_O_2_ for 20 min. White scale bar, 10 μm.

BODIPY 500/510 is a fluorescent fatty acid analog used as an effective tracer of lipid trafficking in oocytes and other cells [14, 37–40]. The numbers, color tone, and distribution pattern of lipid droplets are associated with oocyte quality in mammals [41, 42]. Cellular changes that occur during aging are caused by many different stimuli, and oxidative stress is one of them [22, 43–46]. In addition to young and aged oocytes, we set up additional group of young oocytes treated with H_2_O_2_ to examine the effect of acute exogenous oxidative stress on lipidome. We applied BODIPY 500/510 and CellMask fluorescence staining in young, H_2_O_2_-treated, and aged oocytes at Metaphase II (MII) stage to compare the status of the intracellular natural lipids and plasma membrane. As shown in Fig 1, young oocytes show evenly distributed BODIPY-positive green droplets within the ooplasm, and CellMask shows uninterrupted ring-shaped staining at the oocyte periphery. In contrast, the dramatic difference in both the number of green droplets and the pattern of CellMask staining are noted in aged oocytes. In aged oocytes, CellMask staining at the periphery was often discontinuous with overall low intensity. Green signals were also much reduced in this group (Fig 1). H_2_O_2_-treated oocytes showed somewhat reduced staining of BODIPY 500/510, but CellMask staining seems comparable to young oocytes. These results suggest that both intracellular lipids and the plasma membrane are affected by aging in mouse oocytes.

### The overall workflow for mining differently regulated lipids (DRLs)

**Fig 2:**
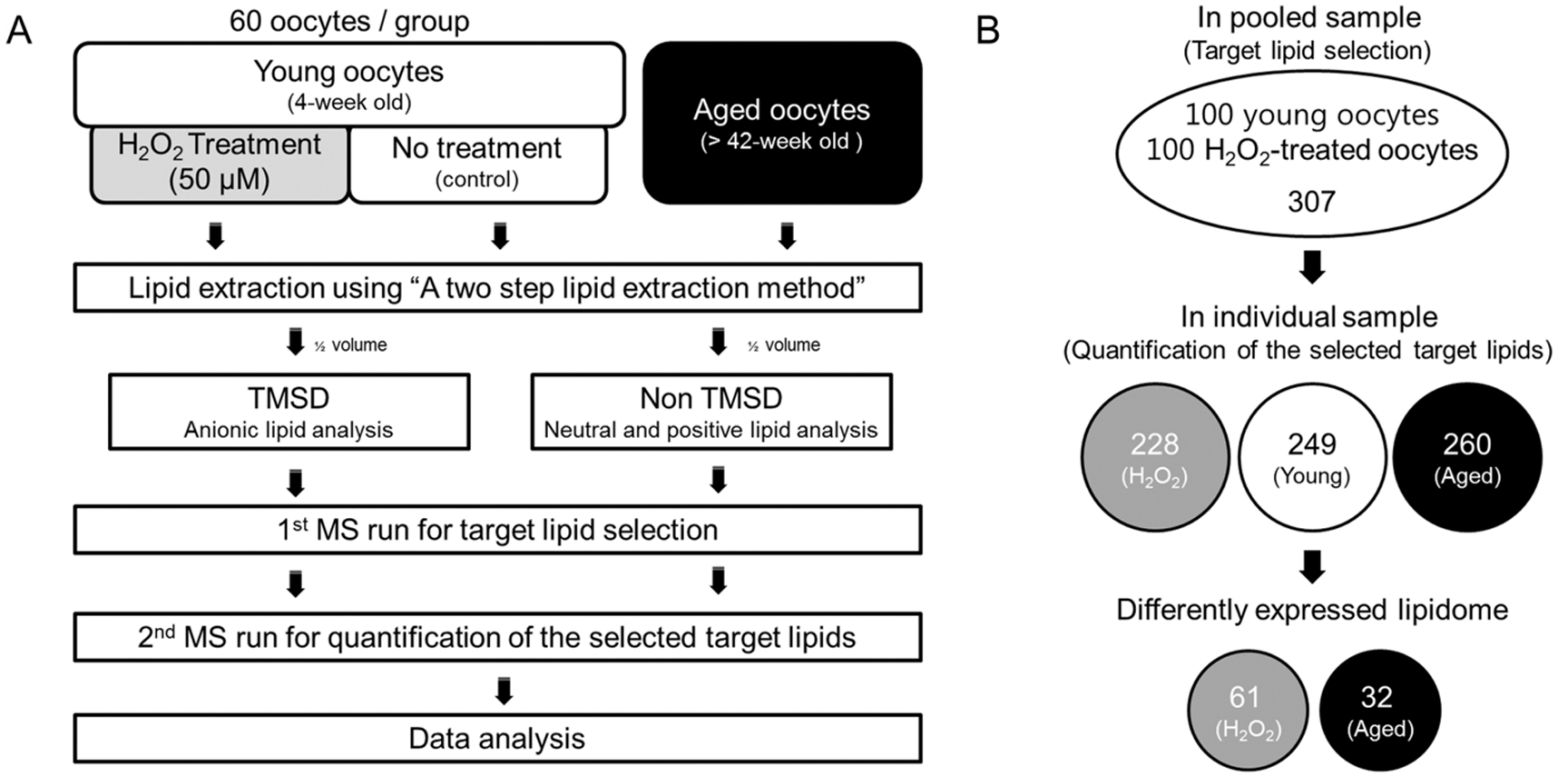
Workflow for lipidomics analysis in three different oocyte samples. (A) A schematic diagram showing the workflow. Three groups of samples were prepared: 60 MII oocytes from 4-week-old ICR mice (young oocytes), 60 H_2_O_2_-treated oocytes from 4-week-old mice (H_2_O_2_-treated), and 60 oocytes from 40-week-old mice or older (aged oocytes). Each sample was subjected to lipid extraction. One half of each extract was methylated with TMSD for anionic lipid detection, and the other half used for non-TMSD detection for neutral- and positive-lipid analysis. Samples were then pooled and analyzed by MS to profile oocyte lipids. The identified peaks [signal-to-noise ratio (S/N) > 3] were selected as target MRMs for quantitative analysis of oocyte lipids in three experimental conditions. To mine differentially regulated lipids (DRLs), data were normalized with internal standards and validated using Student’s *t*-test. (B) This panel shows the process of DRL mining in oocytes. First, 200 oocytes from 4-week-old mice (100 with H_2_O_2_ treatment and 100 untreated) were pooled and analyzed using MS to identify detectable target lipids (S/N > 3) in MII oocytes. From this run, we selected 307 lipid peaks as target transition. In the main experiment, three groups (A) were each subjected to lipid extraction and MS analysis in triplicate with three these target MRM transitions. Among 307 lipids, 249 (S/N > 10) were quantifiable in young oocytes, 260 in aged oocytes, and 228 in H_2_O_2_-treated oocytes. After data validation, we selected 61 and 32 DRLs in H_2_O_2_-treated and aged oocytes, respectively, compared to young oocytes (control). These DRLs are shown in Tables 1 and 2.

We set up three groups of mouse oocytes as shown in Fig 2A to assess lipidome changes associated with oxidative stress and aging: young, H_2_O_2_-treated, and aged oocytes. The number of oocytes in each experimental group was 60 and each group was prepared from several mice to reduce biological variation. After lipid extraction, to find target lipids, global lipid profiling was performed with calculated lipid multiple reaction monitoring (MRM) pairs (in-house database) in pooled lipid mixtures. In this study, 25 lipid classes, namely, four neutral lipids (TG, diacylglycerol [DG], cholesterylester [ChE], cholesterol), six phospholipids (phosphatidylcholine [PC], phosphatidylethanolamine [PE], phosphatidylglycerol [PG], PI, PA, PS), six lysophospholipids (lysophosphatidylcholine [LPC], lysophosphatidylethanolamine [LPE], lysophosphatidylglycerol [LPG], LPI, LPA, LPS), and nine sphingolipids (sphingomyelin [SM], ceramide [Cer], dihydroceramide [dCer], ceramide-1-phosphate [Cer1P], dihydroceramide-1-phosphate [dCer1P], sphingosine [SO], sphinganine [SA], sphingosine-1-phosphate [SO1P], and sphinganine-1-phosphate [SA1P]) were targeted. Validation of the standards used is listed in S1 Table. After global profiling, we found 307 target lipids (S2 Table). For quantitative analysis of the targeted lipidome, we analyzed pooled lipid extracts of oocytes from three different experimental conditions with MRM-MS with target lipid transitions in triplicate. After extracting area information from each raw file by using Skyline software, we selected quantifiable lipid compounds (> 10 signal-to-noise ratio [S/N]). Finally, 32 DRLs were identified in aged oocytes compared to young oocytes (control). For H_2_O_2_-treated oocytes, 61 DRLs were identified (Fig 2B). All DRLs are listed in Tables 1 and 2, and the same lipid species are indicated in bold text. Of the 26 downregulated lipid species in aged oocytes, 19 overlap with those in H_2_O_2_-treated oocytes. Unique lipids in each group are listed in S3 Table.

**Table 1:** Differentially regulated lipids between aged and young oocytes. Fold change ≥ 1.5 (*p* < 0.05).

Upregulated lipids in aged oocytes (6)					Downregulated lipids in aged oocytes (26)				
*Assignment*	*Precursor Ion*	*Product Ion*	*p-value*	Ratio*	*Assignment*	*Precursor Ion*	*Product Ion*	*p-value*	Ratio*
TG(56:7)	922.79	577.5	0.004	2.162	PI(36:0)**	881.6	607.3	0.043	0.213
**PE(36:4)**	740.4	599.3	0.007	1.607	LPE(18:2)	478.3	337.2	0.000	0.242
hE(20:4)	690.6	369.2	0.009	1.588	PI(32:0)**	825.6	551.3	0.000	0.272
**PC(36:4)**	782.5	183.9	0.018	1.851	PI(34:0)**	853.6	579.3	0.033	0.291
**PC(36:5)**	780.5	183.9	0.024	1.959	PI(34:1)**	851.6	577.3	0.000	0.324
**PC(38:5)**	808.5	183.9	0.046	1.548	PI(36:2)**	877.6	603.3	0.033	0.327
					PI(34:2)**	849.6	575.3	0.008	0.350
					TG(60:3)	986.82	603.5	0.048	0.354
					PA(28:5)**	611.5	485.3	0.004	0.425
					LPS(18:0)**	554.3	341.2	0.029	0.444
					PA(30:1)**	647.5	521.3	0.017	0.479
					DG(34:0)	614.5	579.5	0.026	0.494
					PA(28:4)**	613.5	487.3	0.002	0.511
					dCer1P(d14:1–20:5)**	638.5	210.1	0.001	0.511
					PA(30:3)**	643.5	517.3	0.030	0.524
					PA(30:0)**	649.5	523.3	0.001	0.531
					PA(32:3)**	671.5	545.3	0.000	0.541
					DG(32:0)	586.5	551.5	0.020	0.568
					**SO(d14:1)**	244.3	208.1	0.003	0.577
					PE(36:3)	742.4	601.3	0.008	0.579
					LPC(18:2)	520.1	183.9	0.022	0.581
					PS(28:0)**	708.6	495.3	0.005	0.588
					LPS(22:6)**	598.3	385.2	0.009	0.595
					LPS(18:1)**	552.3	339.2	0.016	0.632
					PS(36:1)**	818.6	605.3	0.022	0.638
					Cer1P(d18:1–16:1)**	644.5	264.1	0.004	0.654

* Ratio, aged oocytes/young oocytes

** Acidic lipids analyzed after TMSD derivatization.

**Table 2:** Differentially regulated lipids in H_2_O_2_-treated young oocytes. Fold change ≥ 1.5 (*p* < 0.05).

Upregulated lipids in H_2_O_2_-treated oocytes (26)					Downregulated lipids in H_2_O_2_-treated oocytes (35)				
*Assignment*	*Precursor Ion*	*Product Ion*	*p-value*	Ratio*	*Assignment*	*Precursor Ion*	*Product Ion*	*p-value*	Ratio*
DG(36:0)	642.6	607.6	0.00	41.73	PI(36:0)**	881.6	607.3	0.03	0.12
DG(34:0)	614.5	579.5	0.00	25.51	PI(34:1)**	851.6	577.3	0.00	0.13
DG(32:0)	586.5	551.5	0.00	17.29	LPI(18:0)**	615.2	341.1	0.01	0.13
DG(30:0)	558.5	523.5	0.01	3.77	PI(34:0)**	853.6	579.3	0.02	0.13
PC(42:6)	862.5	183.9	0.01	3.15	PI(32:0)**	825.6	551.3	0.00	0.14
DG(36:7)	628.4	593.4	0.05	2.28	PI(34:2)**	849.6	575.3	0.00	0.15
PC(36:0)	790.5	183.9	0.02	2.06	PI(36:2)**	877.6	603.3	0.02	0.16
PC(38:3)	812.5	183.9	0.01	2.02	DG(32:1)	584.5	549.5	0.03	0.22
PC(38:6)	806.5	183.9	0.01	1.91	PE(32:1)	690.4	549.3	0.02	0.24
PC(36:2)	786.5	183.9	0.00	1.88	LPS(18:0)**	554.3	341.2	0.02	0.25
PC(38:2)	814.5	183.9	0.05	1.85	SM(d18:1–18:1)	729.3	183.9	0.01	0.29
LPA(18:1)**	465.3	339.2	0.00	1.80	PA(30:1)**	647.5	521.3	0.01	0.36
PC(34:1)	760.5	183.9	0.00	1.78	PA(32:3)**	671.5	545.3	0.00	0.37
**PE(36:4)**	740.4	599.3	0.01	1.76	PS(36:1)**	818.6	605.3	0.00	0.37
PC(34:2)	758.5	183.9	0.03	1.71	PA(28:1)**	619.5	493.3	0.03	0.37
PC(36:1)	788.5	183.9	0.04	1.70	LPS(22:6)**	598.3	385.2	0.00	0.38
PE(36:2)	744.4	603.3	0.00	1.68	SM(d18:1–22:0)	787.3	183.9	0.03	0.40
**PC(38:5)**	808.5	183.9	0.04	1.68	PA(28:4)**	613.5	487.3	0.00	0.40
**PC(36:5)**	780.5	183.9	0.01	1.64	PA(30:0)**	649.5	523.3	0.00	0.41
PC(34:0)	762.5	183.9	0.02	1.62	PA(30:3)**	643.5	517.3	0.03	0.43
PC(32:0)	734.5	183.9	0.02	1.61	PA(28:5)**	611.5	485.3	0.02	0.43
PE(36:1)	746.4	605.3	0.04	1.61	PS(36:2)**	816.6	603.3	0.03	0.48
**PC(36:4)**	782.5	183.9	0.02	1.58	LPS(18:1)**	552.3	339.2	0.01	0.53
PE(38:4)	768.4	627.3	0.03	1.55	SM(d18:1–18:0)	731.3	183.9	0.04	0.53
PG(36:3)	790.4	601.3	0.05	1.53	Cer1P(d18:1–16:0)**	646.5	264.1	0.01	0.56
PE(36:3)	742.4	601.3	0.03	1.50	PS(28:0)**	708.6	495.3	0.01	0.59
					LPA(18:3)**	461.3	335.2	0.00	0.60
					ChE(18:1)	668.6	369.2	0.00	0.60
					**SO(d14:1)**	244.3	208.1	0.00	0.61
					Cer1P(d18:1–16:1)**	644.5	264.1	0.01	0.61
					SM(d18:1–14:0)	675.3	183.9	0.01	0.63
					LPI(14:0)**	559.2	285.1	0.00	0.63
					ChE(22:6)	714.6	369.2	0.00	0.64
					LPC(20:4)	544.1	183.9	0.01	0.67
					SM(d18:1–24:1)	813.3	183.9	0.01	0.67

* Ratio, aged oocytes/young oocytes.

** Acidic lipids analyzed after TMSD derivatization.

### The alteration of all detected lipid species

**Fig 3:**
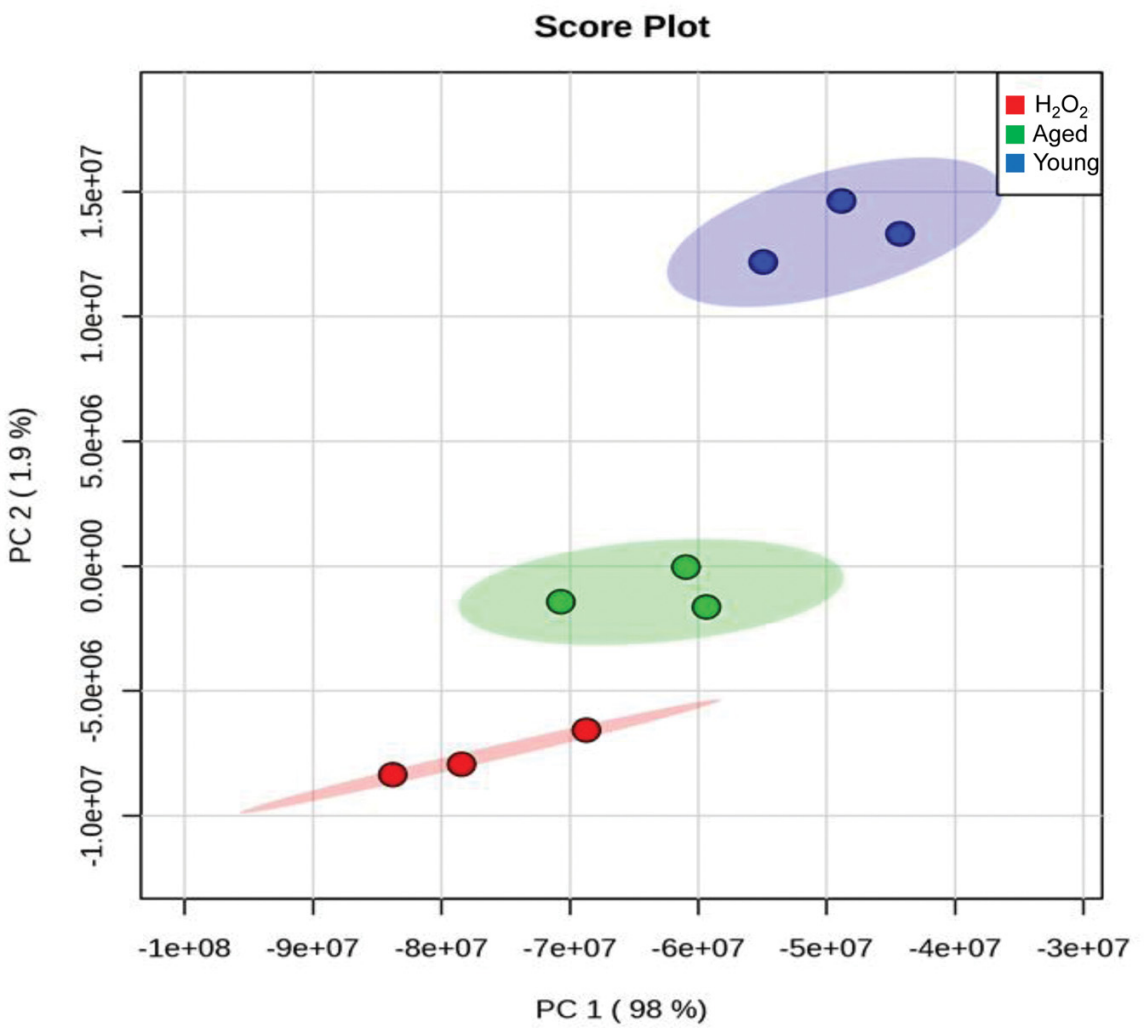
Principal component analysis (PCA) plot for MS analysis of young, aged, and H_2_O_2_-treated oocyte lipids. Young oocytes, blue dots; aged oocytes, green dots; H_2_O_2_-treated oocytes, red dots.

**Fig 4:**
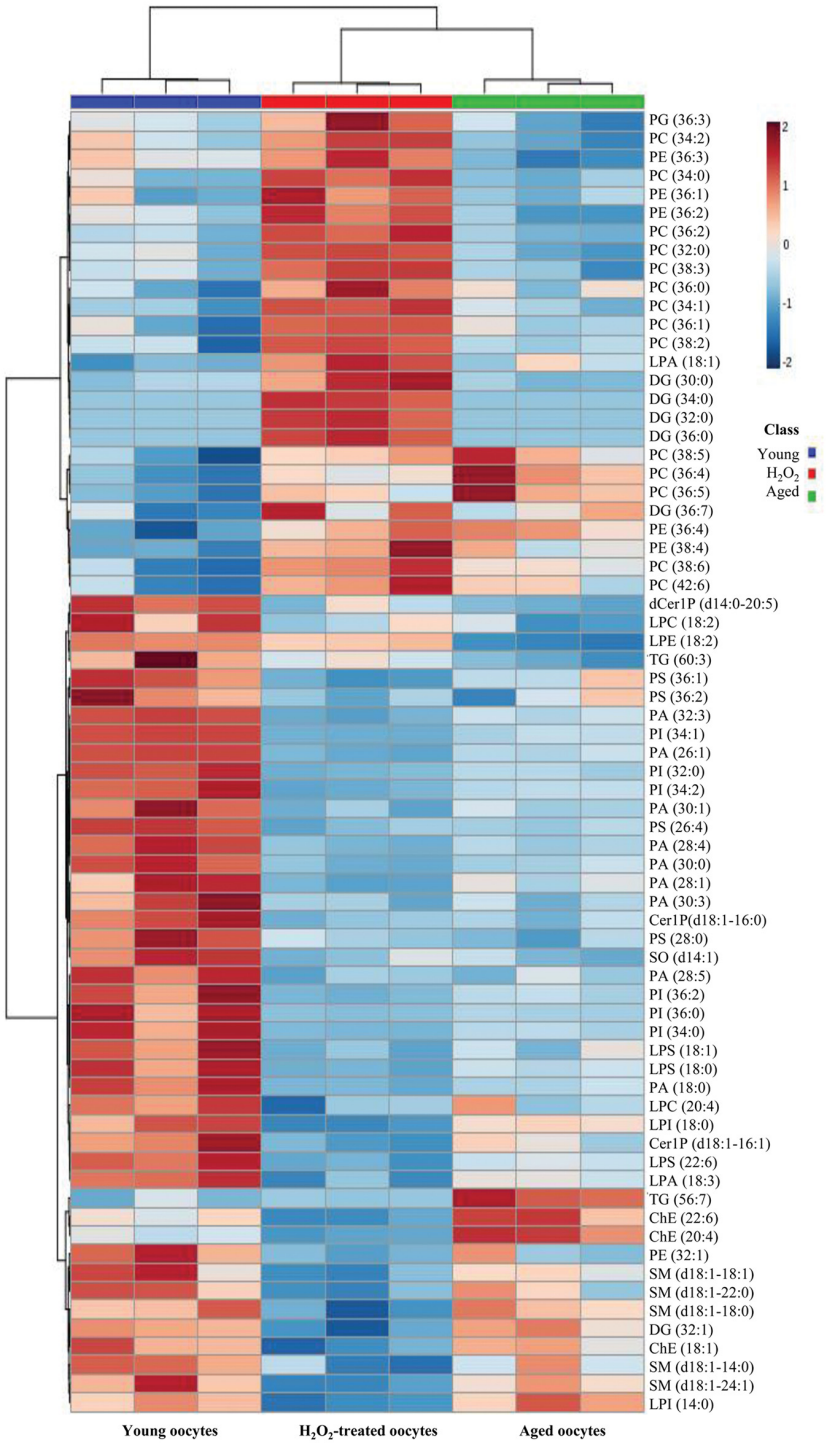
Hierarchical clustering of datasets from triplicate MS analysis showing differentially regulated lipids in young, H_2_O_2_-treated, and aged MII oocytes.

**Fig 5:**
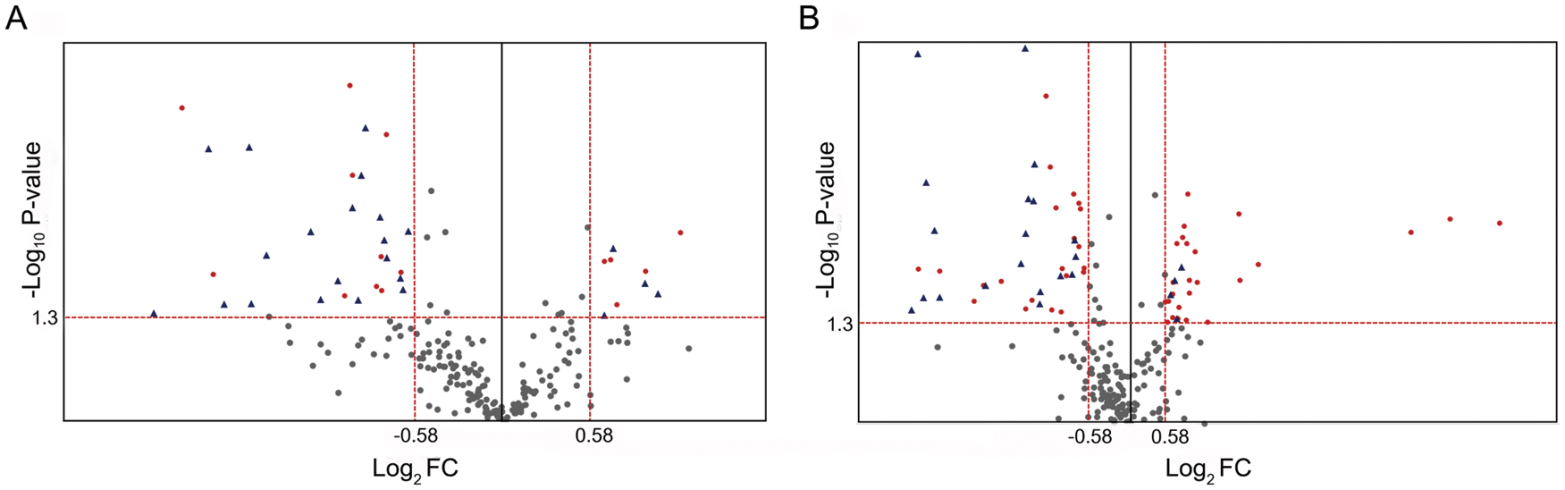
The volcano plots of detected lipids of each set of compared groups. Grey dots indicate lipids showing no differences in two sets of compared groups. (A) Red dots indicate DRLs in aged oocytes. (B) Red dots indicate DRLs in H_2_O_2_-treated oocytes. Red dots indicate detected lipid species with fold changes greater than 1.5 (*p* < 0.05). Blue triangles in both plots indicate common DRLs between two compared groups. FC, fold change.

We next performed principal component analysis (PCA) with total MRM datasets. As shown in Fig 3, three groups were well separated. The distance between H_2_O_2_-treated and aged oocytes was closer than that of young oocytes, and this may imply that certain lipids show similar tendencies in H_2_O_2_-treated and aged oocytes. Next, we performed hierarchical cluster analysis of the acquired MRM spectra (Fig 4). It also clearly showed that the datasets were well separated and easily distinguishable among young, aged, and H_2_O_2_-treated oocytes. The differentially regulated lipids are represented in volcano plots, in which the level of lipids from aged or H_2_O_2_-treated oocytes was compared to those of young oocytes (Fig 5). The result shows that many downregulated lipids overlap between old and H_2_O_2_-treated oocytes while upregulated lipids were unique to aged and H_2_O_2_-treated oocytes (Fig 5, blue triangles).

### Correlation of changes in lipid classes

**Fig 6:**
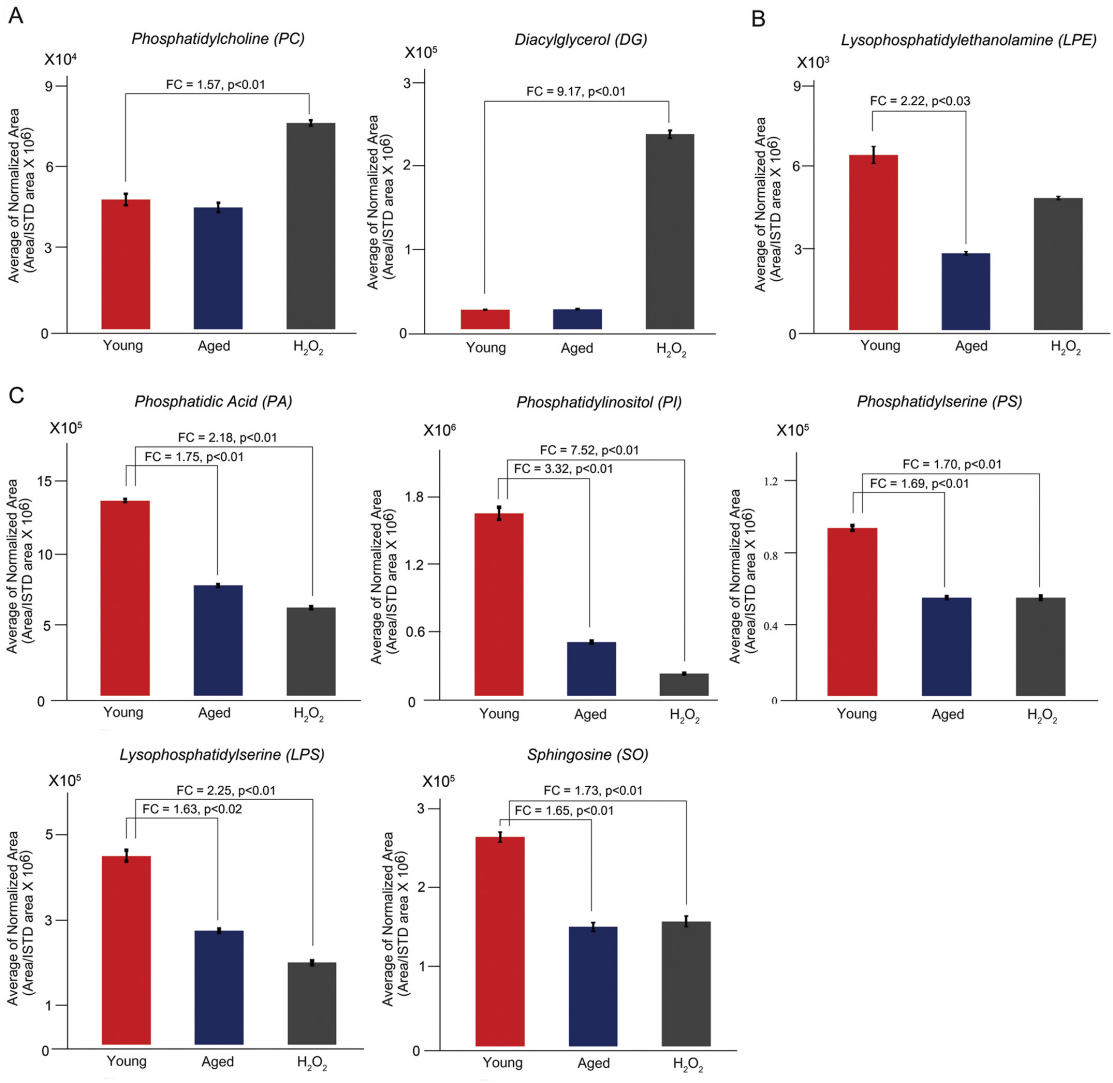
Lipid classes that are significantly changed between two sets of compared groups. (A) Upregulated lipid classes and (B) downregulated lipid classes in comparison with young oocytes (control). Some of the downregulated lipid classes are similar in aged and H_2_O_2_-treated oocyte groups, but no significantly increased lipid class was identified in aged oocytes. Fold changes greater than 1.5 are shown (*p* < 0.05). ISTD, internal standard.

Because many lipid classes have common structural features and are often regulated by the same enzymes in class-specific manners, a high degree of co-regulation is presumed in oocyte lipid profiles. Therefore, we analyzed the class-specific level of lipid alteration. We selected the lipid classes that showed greater than 1.5-fold changes (*p* < 0.05) against young oocyte lipid levels. Lipid classes that were not significantly changed among the groups are shown in S1 Fig. Two lipid classes, PC and DG, were identified as significantly increased only in H_2_O_2_-treated oocytes (Fig 6A). However, no specific lipid class was significantly increased only in aged oocytes. LPE was significantly decreased only in aged oocytes (Fig 6B), while PA, PI, PS, LPS, and SO were downregulated both in H_2_O_2_-treated and aged oocytes (Fig 6C). PI showed the highest degree of alteration, i.e., 7.52-fold and 3.32-fold decreases in H_2_O_2_-treated and aged oocytes, respectively. Thus, phospholipids were the most vulnerable lipid classes in both groups. Consistent with previous studies showing that phospholipids are affected by oxidative stress [19, 47, 48], we also observed that phospholipids exhibit the highest degree of weakness to the exogenous oxidative stress condition used herein in mouse oocytes. These results partly suggest that the vulnerability of phospholipids in aged mouse oocytes may involve oxidative stress. However, many other lipid classes showed differing patterns in these groups. Thus, aging-associated changes in lipidome in mouse oocytes is distinct from those induced by an acute oxidative stress.

### Upregulation of lysophosphatidylcholine acyltransferase 1 in H_2_O_2_-treated oocytes

**Fig 7:**
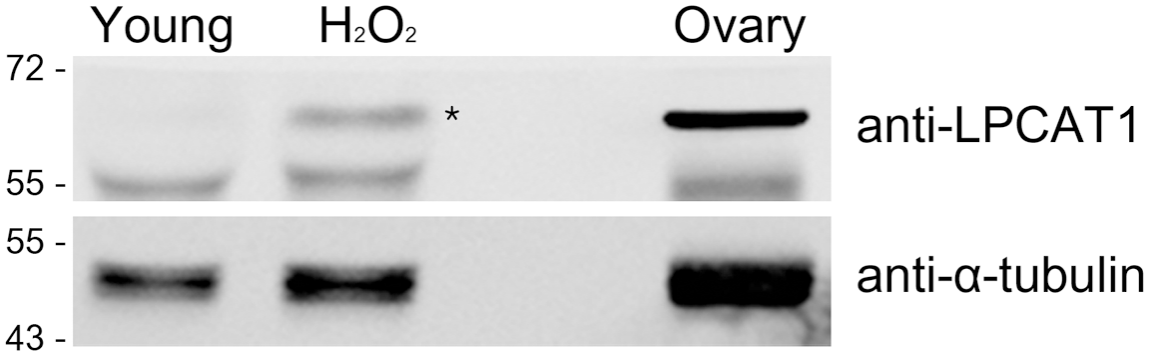
Western blotting of LPCAT1 in H_2_O_2_-treated oocytes. MII oocytes from 4-week-old mice were oxidized with 50 μM H_2_O_2_ for 20 min. Control oocytes were incubated in PBS. Oocytes (100 oocytes each) were directly collected in 12 μL of 1× sample buffer and run in a 10% SDS-PAGE gel. After transfer, western blotting was performed with anti-LPCAT1 and anti-α-tubulin primary antibodies. Ovarian extract was used as a positive control. The asterisk indicates LPCAT1 product.

In our lipidome analysis, we added a group of H_2_O_2_-treated oocytes to examine if exogenous oxidative stressor can mimic aging process with respect to the alteration of lipidome. As mentioned above, several classes of phospholipids were similarly affected in H_2_O_2_-treated and aged oocytes, but others showed differing patterns. A notable change in H_2_O_2_-treated oocytes is a dramatic increase in DG (Fig 6A), which is the second signaling messenger that activates the protein kinase C (PKC) pathway [49]. Decreases in PI along with increases in DG suggest that PI is hydrolyzed to produce DG and phosphatidylinositol triphosphate (PIP_3_), subsequently activating calcium release and PKC [49]. Indeed, it has been shown that H_2_O_2_ treatment of mammalian oocytes increases intracellular calcium content [50, 51]. Oxidative stress can also lead to the accumulation of free fatty acids, which causes lipotoxicity in cells [52]. These free fatty acids can be metabolized by lysophosphatidylcholine acyltransferase 1 (LPCAT1) to PC [53]. In our analysis, the PC class significantly increased only in H_2_O_2_-treated oocytes (Fig 6A). Thus, we hypothesized that increased DG in H_2_O_2_-treated oocytes activates cytosolic phospholipase A2α (cPLA2α) in oocytes, which causes lipotoxicity by releasing free fatty acids, and this in turn is neutralized by activation of LPCAT1 in this group. To test this hypothesis, we performed western blotting of cPLA2α and LPCAT1 in young and H_2_O_2_-treated oocytes. In MII oocytes (100 oocytes), cPLA2 was undetectable by western blotting (data not shown). The level of LPCAT1 increased in H_2_O_2_-treated oocytes as shown in Fig 7. This result suggests that the LPCAT1 system may serve to neutralize lipotoxicity during H_2_O_2_-induced oxidative stress.

## Discussion

Lipids are used as structural backbones as well as energy sources in mammalian oocytes [16], and their alteration affects developmental competence [12–14]. Aging clearly decreases fertilization and developmental potentials in oocytes, but the underlying cause of such negative influence is uncertain. Qualitatively, CellMask staining of aged oocytes showed partial disruption of peripheral staining, and the number of BODIPY 500/510-positive intracellular dots decreased noticeably (Fig 1). By MS analysis, we found that several classes of phospholipids quantitatively decreased in aged oocytes. PA, PI, PS, and LPS classes similarly showed decreased pattern both in H_2_O_2_-treated and aged oocytes, suggesting that these lipids may be the targets of oxidative stress during aging (Fig 6C). However, H_2_O_2_ treatment in our study is given as an exogenous acute insult which is fundamentally different from mitochondria-originated oxidative stress considered as a cause of cellular aging. H_2_O_2_ treatment dramatically increases DG in oocytes, as shown in Fig 6A. However, aging did not increase DG levels in oocytes (Fig 6A). H_2_O_2_ possibly mounted a full-fledged and exaggerated signaling cascade as an acute response, which is generally not observed under normal or aging conditions. H_2_O_2_ treatment has been shown to induce phosphorylation of cPLA2 via the activation of extracellular-regulated kinase 1/2, thereby increasing free fatty acids, which causes lipotoxicity [52]. To reduce lipotoxicity, lipid remodeling processes involving specific enzymes would be operative, which results in increases in PC and PE lipid classes. Western blot analysis revealed that the level of LPCAT1 in oocytes increases after H_2_O_2_ treatment, suggesting that LPCAT1 functions to reduce lipotoxicity caused by oxidative stress. Since the DG and PC classes did not increase in aged oocytes, aged oocytes do not seem to experience such a notable effect of phospholipid breakdown (Fig 6).

Our analyses also showed that all detected PI species, i.e., PI(36:0), (32:0), (34:0), (34:1), (36:2), and (34:2), were downregulated in both aged and H_2_O_2_-treated groups. This observation indicates potential breakdown of PI species, which generally leads to production of PIP_3_ and DG. As has been widely shown in many systems, increases in PIP_3_ and DG normally lead to the activation of the phosphoinositide 3-kinase (PI3K) pathway [54]. However, in contrast to dramatic increases in DG in H_2_O_2_-treated oocytes, there was no significant increase in DG in aged oocytes. A possible explanation for this observation was provided by Takekoshi et al., who demonstrated that oxidative DG could also activate the PI3K pathway [55]. Since we did not add MRM pairs of oxidized DG in the initial run, we were not able to detect this form of DG.

Due to limited availability of aged oocyte samples, several aspects of potential lipotoxicity were not addressed in the present investigation. Whether oxidized DG levels increase in aged oocytes and whether the activation of cPLA2 and PI3K signaling cascades is associated with phospholipid breakdown under oxidative stress in oocytes requires further investigation.

## Materials and Methods

### Oocyte collection

Mice were maintained in accordance with the policies of the Konkuk University Institutional Animal Care and Use Committee (IACUC). This study was approved by the Konkuk University IACUC (approval number KU12081). Four-week-old ICR mice and 6-month-old ICR mice were purchased from Orient-Bio (Gyeonggi-do, Korea) and were housed in a controlled barrier facility at Konkuk University. The mouse room is equipped with an automated light-dark cycle system and mice were fed a general diet (5L79 diet, LabDiet, St. Louis, MO, USA). Four-week-old mice were superovulated by injecting 7.5 IU pregnant mare serum gonadotropin (PMSG; Sigma-Aldrich, St. Louis, MO, USA) and 7.5 IU human chorionic gonadotropin (hCG) at 48-h intervals, and aged female mice (42–50 weeks old) were injected with 20 IU PMSG and hCG [56]. Young mice ovulated average 15–20 oocytes at 7.5 IU, whereas aged mice did not ovulate. At higher doses aged mice ovulated average 3–5 oocytes. Oocytes without any obvious deformation or discoloration were selected for further analysis. Thirteen hours after hCG injection, mice were sacrificed under anesthesia and COCs were collected by oviduct flushing. Cumulus cells were removed by treating COCs with hyaluronidase (300 μg/mL) for 2 min. MII oocytes were collected, transferred to Quinn’s Advantage medium with *N*-2-hydroxyethylpiperazine-*N′*-2-ethanesulfonic acid (HEPES, Cooper Surgical, Trumbull, CT, USA) containing 20% fetal bovine serum (Ref # 12483–020, lot # 1129906, Life Technologies, Grand Island, NY, USA), and cultured at 37°C.

### H_2_O_2_ treatment

MII oocytes from 4-week-old mice were separated into two groups, one of which was incubated in 50 μM H_2_O_2_ for 20 min **to provide exogenous oxidative stress [50, 57, 58]**. Oocytes were then washed with media three times and prepared along with the other groups.

### Confocal live imaging

Young, H_2_O_2_-treated, and aged oocytes were treated with two fluorescent dyes to visualize natural fatty acids and plasma membrane [40]. To stain oocyte plasma membranes, CellMask Deep Red Plasma Membrane Stain (C10046, Invitrogen, Grand Island, NY, USA) was used at 2.5 μg/mL in media for 30 min. BODIPY fatty acid 500/510 (D-3823, Invitrogen) was used for staining natural fatty acids in mouse oocytes. These oocytes were washed three times with media and transferred to a glass-bottom confocal dish (SPL Life Sciences, Pocheon, Korea). Live-cell images were captured with an Olympus FV1000 spectral confocal microscope (Olympus, Tokyo, Japan) equipped with a warm plate or with an LSM 710 confocal microscope (Carl Ziess, Oberkochen, Germany).

### Reagents

HPLC-grade acetonitrile, methanol, water, and 2-propanol were purchased from J.T. Baker (Avantor Performance Material, Inc., Center Valley, PA, USA). Fluka Analytical HPLC-grade formic acid, chloroform, ammonium formate, and trimethylsilyldiazomethane (TMSD) were purchased from Sigma-Aldrich. All lipid standards (Avanti Polar Lipids, Inc., Alabaster, AL, USA) used in this study were as follows: phosphatidylcholine (PC (10:0–10:0), PE (10:0–10:0), PG (10:0–10:0), PI (8:0–8:0), PS (10:0–10:0), PA (10:0–10:0), LPC (C13:0), LPE (C14:0), LPG (C14:0), LPI (C13:0), LPS (C17:1), lysophosphatidic LPA (C17:0), SM (d18:1–12:0), Cer (d18:1–12:0), dCer (d18:0–12:0), SO (C17:1), SA (C17:0), Cer1P (d18:1–12:0), dCer1P (d18:0–16:0), SO1P (C17:1), and SA1P (C17:0). TG (11:1–11:1–11:1), DG (8:0–8:0), and ChE (10:0) were purchased from Larodan Fine Chemicals AB (Malmö, Sweden).

### Sample Preparation

Each lipid standard was dissolved in chloroform/methanol (1:1, v/v) and stored at –20°C. To extract more anionic lipids, oocyte lipids were subject to two-step extractions that can disrupt ionic interactions between acidic lipids and proteins. Firstly, extracted oocytes (n = 60 for each group) were added to 990 μL of chloroform/methanol (1:2, v/v) and 10 μL of a 1 μg/mL solution of the commercial lipids listed above as internal standards. Samples were subsequently vortexed 3 × 30 s and incubated at room temperature for 10 min. After centrifugation (13,800 × *g*, 2 min at 4°C), approximately 1 mL of supernatant was transferred to a 1.5- mL tube. Secondly, the remaining pellets were dissolved in 750 μL chloroform/methanol/37% HCl (40:80:1, v/v/v) and incubated at room temperature for 15 min with vortexing 3 × 30 s. Then, 250 μL of cold chloroform and 450 μL of cold 0.1 N HCl were added. The samples were vigorously vortexed and then centrifuged at 6,500 × *g* for 2 min at 4°C. The bottom organic phase was collected and two collected lipid mixes were pooled [59]. Subsequently, the samples were divided into two equal aliquots and dried in a SpeedVac concentrator. One of the dried samples was then redissolved in 50 μL of solvent A/solvent B (2:1, v/v) for neutral and positive lipid analysis, and the other one was reconstituted in 50 μL of methanol for the TMSD methylation reaction and anionic lipid analysis. Solvent A consisted of acetonitrile–methanol–water (19:19:2) with 20 mM ammonium formate and 0.1% (v/v) formic acid, and solvent B consisted of 2-propanol with 20 mM ammonium formate and 0.1% (v/v) formic acid.

### TMSD methylation

For analysis of acidic lipids that are difficult to analyze by LC-MS because of peak tailing, we used a TMSD methylation method [28]. A 2 M solution of TMSD in 50 μL hexane was added to the lipid extracts resuspended in 50 μL of methanol. After vortexing for 30 s, methylation was performed at 37°C for 15 min (optimized conditions). Addition of 6 μL of glacial acetic acid quenched the methylation for subsequent analysis.

### Global lipid analysis of oocyte samples by using HPLC/MS

The HPLC analysis was performed on an Agilent 1290 infinity series HPLC instrument (Agilent Technologies, Santa Clara, CA, USA) equipped with binary pump (G20A), an autosampler (G4226A), a column compartment (G1316C) and a thermostat (G1330B). Hypersil GOLD column (2.1 × 100 mm ID; 1.9 μm, Thermo Fisher Scientific, Waltham, MA, USA) was used for the separation of lipids. The temperature of column oven and sample tray was adjusted to 40°C and 4°C, respectively. Solvent A consisted of a acetonitrile/methanol/water mixture (19:19:2) with 0.1% (v/v) formic acid and 20 mM ammonium formate, and solvent B consisted of 2-propanol with 0.1% (v/v) formic acid and 20 mM ammonium formate. The flow rate was 0.25 mL/min and the injection volume was 2 μL for each run. A 30-min lipid elution gradient was performed as follows: during the first 5 min, solvent composition was set at 95% A and 5% B; followed by a first linear gradient to 70% A and 30% B for 10 min; then the solvent ratio was linearly changed to 5% A and 95% B for 7 min and maintained for 3 min. Finally, the column was equilibrated at 5% solvent B for 5 min before reuse. Lipid analysis of oocyte samples was performed by using a triple quadrupole mass spectrometer (QQQ LC-MS 6490 series, Agilent Technologies) equipped with an ESI source which provides high sensitivity by iFunnel technology that consists of three components: a hexabore capillary, Agilent Jet Stream technology, and a dual ion funnel. The typical operating source conditions for MS scan in the positive ion ESI mode were optimized as follows: ESI source settings were as follows: capillary voltage 2500 V; nozzle voltage 500 V. The nebulizer was set at 40 psig and the nitrogen drying gas was set at a flow rate of 13 L/min. Gas and drying gas temperatures were maintained at 200°C/180°C for neutral and positive lipid analysis and 180°C/180°C for TMSD-reacted lipid analysis (optimized condition). All the lipids were recorded under optimized experimental conditions and quantification analysis was performed in MRM mode using computed transitions for each lipid species.

### Data processing and statistical analysis of individual data obtained by MRM

LC/MS data were obtained by Agilent Mass Hunter Workstation Data Acquisition software. The MRM data for target lipids, including *m/z* of precursor ions, *m/z* of product ions, retention time, etc., were exported by Qualitative Analysis B.06.00 software (Agilent Technologies). Rawdata are deposited in Harvard Dataverse site (https://dataverse.harvard.edu/, http://dx.doi.org/10.7910/DVN/C2OOIE). Because lipids are separated based on their fatty acid composition, lipid peaks could be assigned by comparing to the retention time of each class internal standard. Next, for mining area information of each assigned lipid from replicated raw data, Skyline software (MacCoss Lab, University of Washington, Seattle, WA, USA; https://brendanx-uw1.gs.washington.edu/labkey/wiki/home/software/Skyline/events/2013%20User%20Group%20Meeting%20at%20ASMS/page.view?name=hoofnagle) was used with an in-house database. Extracted area information of all lipids was normalized by internal standards based on their classes [5]. Subsequently, the Student's *t*-test was used for the statistical analysis and calculation of fold changes using Microsoft Excel software. Finally, differently expressed lipids were rearranged by the cutoff using a *p* value of 0.05 and fold-change value of 1.5. We also selected lipid species having reasonable fatty acid composition in mammal cells (over 14 carbons). To show the correlation between samples and lipids, hierarchical clustering of the selected lipids was performed using the MetaboAnalyst web site (www.metaboanalyst.ca).

### Western blotting

For each group, 100 MII oocytes were directly collected in 12 μL of 2× sodium dodecyl sulfate (SDS) sample buffer (100 mM Tris-Cl [pH 6.8], 4% SDS, 20% glycerol, 2% β-mercaptoethanol, and 0.2% bromophenol blue). The samples were boiled and centrifuged briefly, and then loaded onto 10% SDS-polyacrylamide gels. After electrophoresis, gels were blotted onto nitrocellulose membranes (Millipore, Billerica, MA, USA). The membranes were then blocked with 5% skim milk in Tris-base saline for 1 h at room temperature and incubated with primary antibodies at 4°C overnight. They were then incubated with peroxidase-conjugated secondary antibodies (GenDEPOT, Barker, TX, USA) diluted 1:10000 for 1 h. Chemiluminescence signals were detected using the LAS4000 system (Fujifilm, Tokyo, Japan). The primary antibodies used were rabbit polyclonal anti-LPCAT1 (1:1000, Proteintech, Chicago, IL, USA) and mouse monoclonal anti-α-tubulin (1:10000, Sigma-Aldrich).

## Supporting Information

S1 Fig Lipid classes that were not significantly changed among experimental groups. (PDF)

S1 Table Validation study of lipid analysis based on multiple reaction monitoring (MRM) and the limit of detection (LOD) of each lipid standard (30 min). (PDF)

S2 Table Detected lipid species in oocytes. (PDF)

S3 Table Lipid species uniquely expressed in each comparison. Data from three technical replicates are shown.
(PDF)
