# Supplementary material for: Age-Associated Lipidome Changes in Metaphase II Mouse Oocytes
Source: PLoS One. 2016 Feb 16;11(2):e0148577. doi: 10.1371/journal.pone.0148577 (PMC4755615; doi:10.1371/journal.pone.0148577)
Supplement: S1 Fig — (PDF) [file pone.0148577.s001.pdf]

S1 Fig. Lipid classes that were not significantly changed among experimental groups.

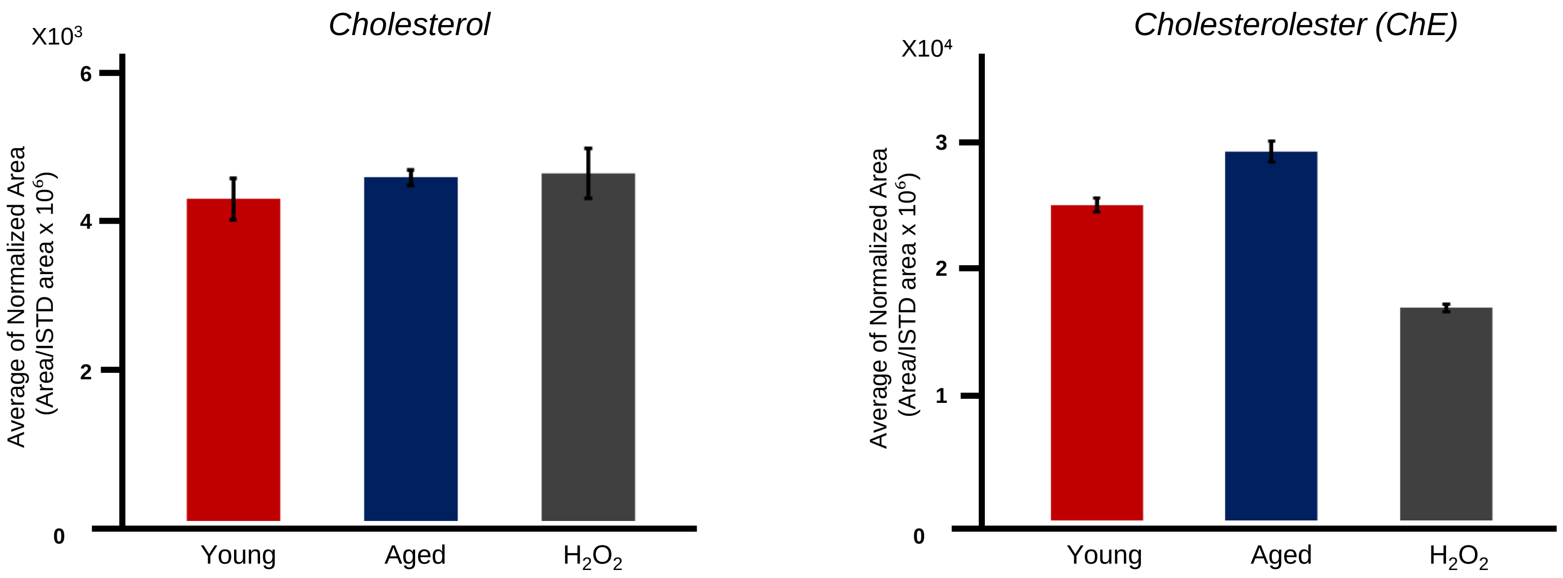

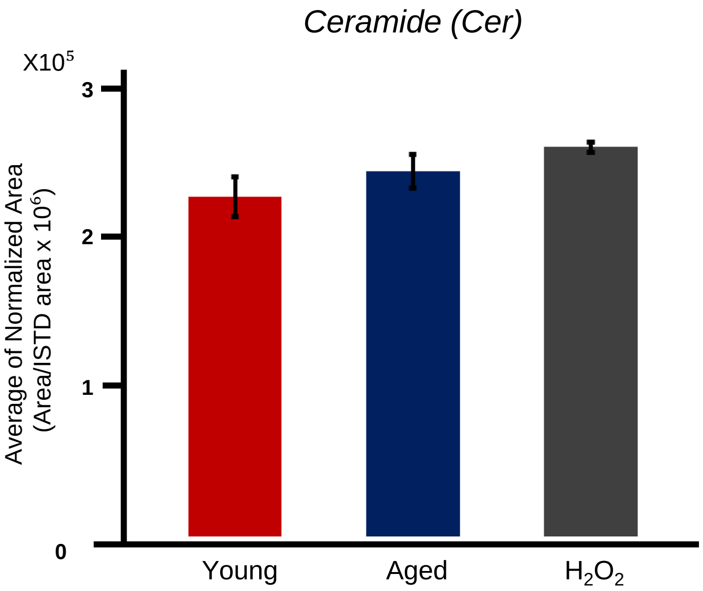
Ceramide (Cer)
X10^5
3
2
1
0
Average of Normalized Area
(Area/ISTD area x 10^6)
Young
Aged
H2O2

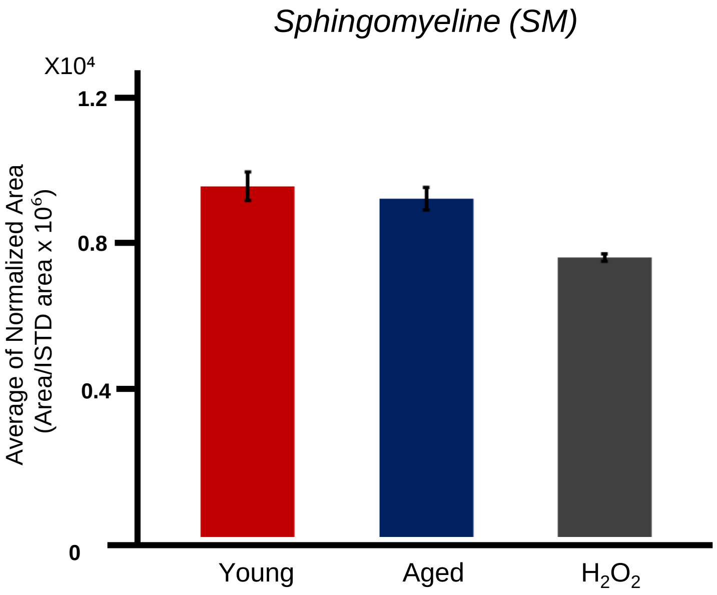
Sphingomyeline (SM)
X10^4
1.2
0.8
0.4
0
Average of Normalized Area
(Area/ISTD area x 10^6)
Young
Aged
H2O2

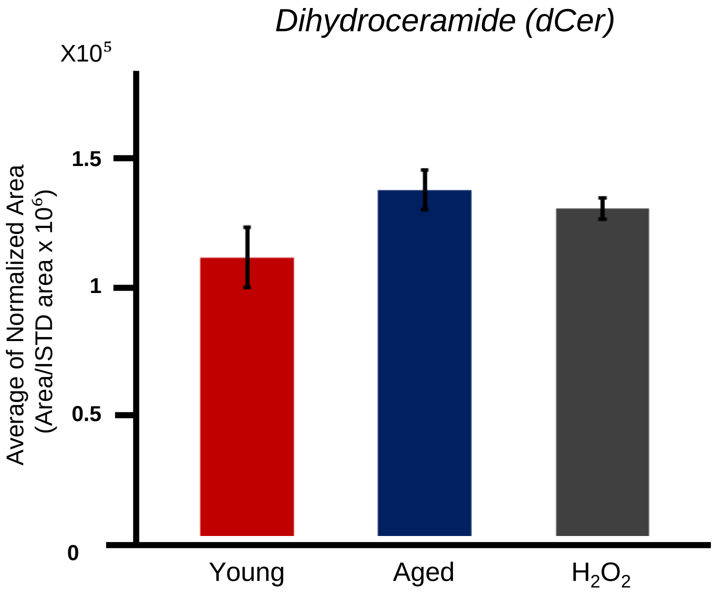
Dihydroceramide (dCer)
X10^5
1.5
1
0.5
0
Average of Normalized Area
(Area/ISTD area x 10^6)
Young
Aged
H2O2

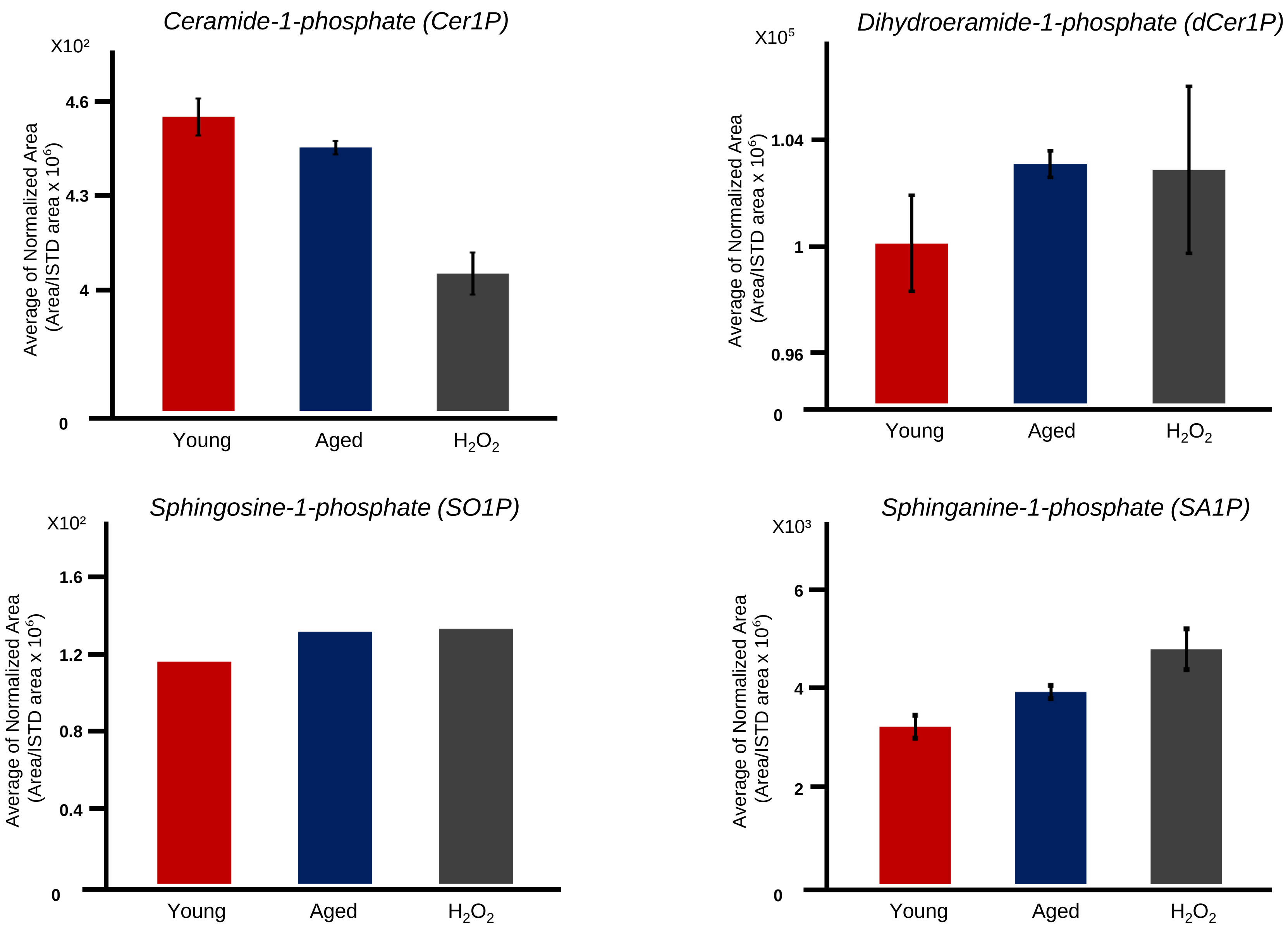

Ceramide-1-phosphate (Cer1P)
X10²
4.6
4.3
4
0
Average of Normalized Area
(Area/ISTD area x 10⁶)
Young
Aged
H2O2
Dihydroeramide-1-phosphate (dCer1P)
X10⁵
1.04
1
0.96
0
Average of Normalized Area
(Area/ISTD area x 10⁶)
Young
Aged
H2O2
Sphingosine-1-phosphate (SO1P)
X10²
1.6
1.2
0.8
0.4
0
Average of Normalized Area
(Area/ISTD area x 10⁶)
Young
Aged
H2O2
Sphinganine-1-phosphate (SA1P)
X10³
6
4
2
0
Average of Normalized Area
(Area/ISTD area x 10⁶)
Young
Aged
H2O2

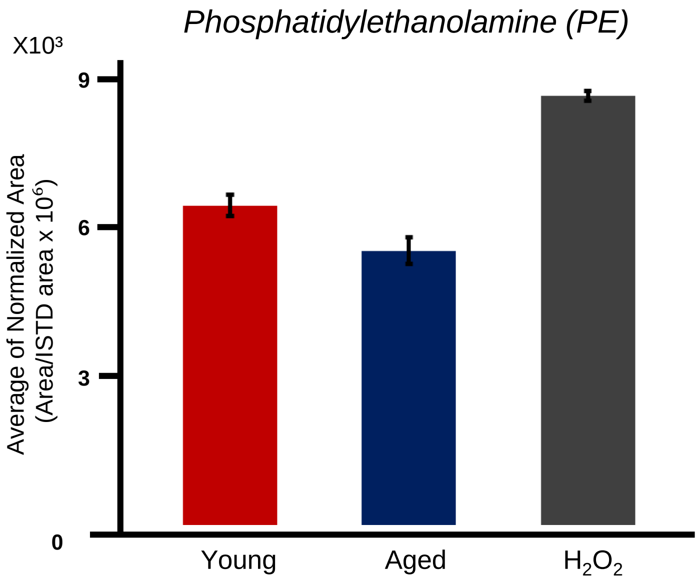
Phosphatidylethanolamine (PE)
X10³
9
6
3
0
Average of Normalized Area
(Area/ISTD area x 10⁶)
Young
Aged
H2O2

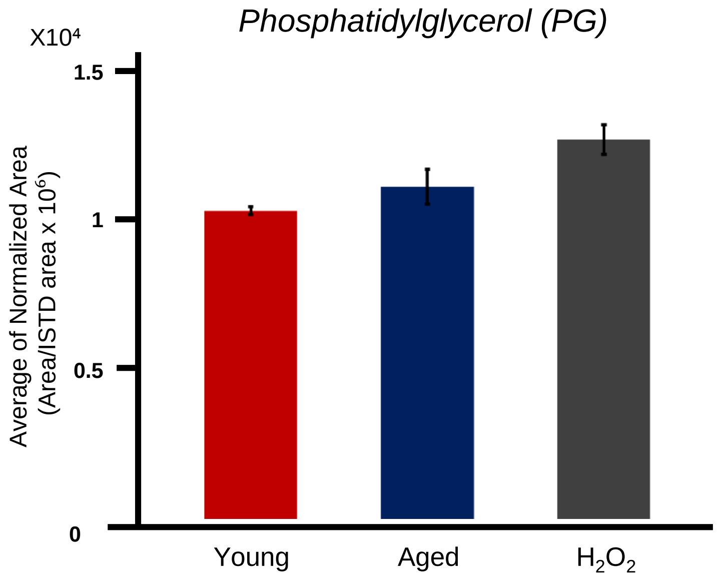
Phosphatidylglycerol (PG)
X10⁴
1.5
1
0.5
0
Average of Normalized Area
(Area/ISTD area x 10⁶)
Young
Aged
H2O2

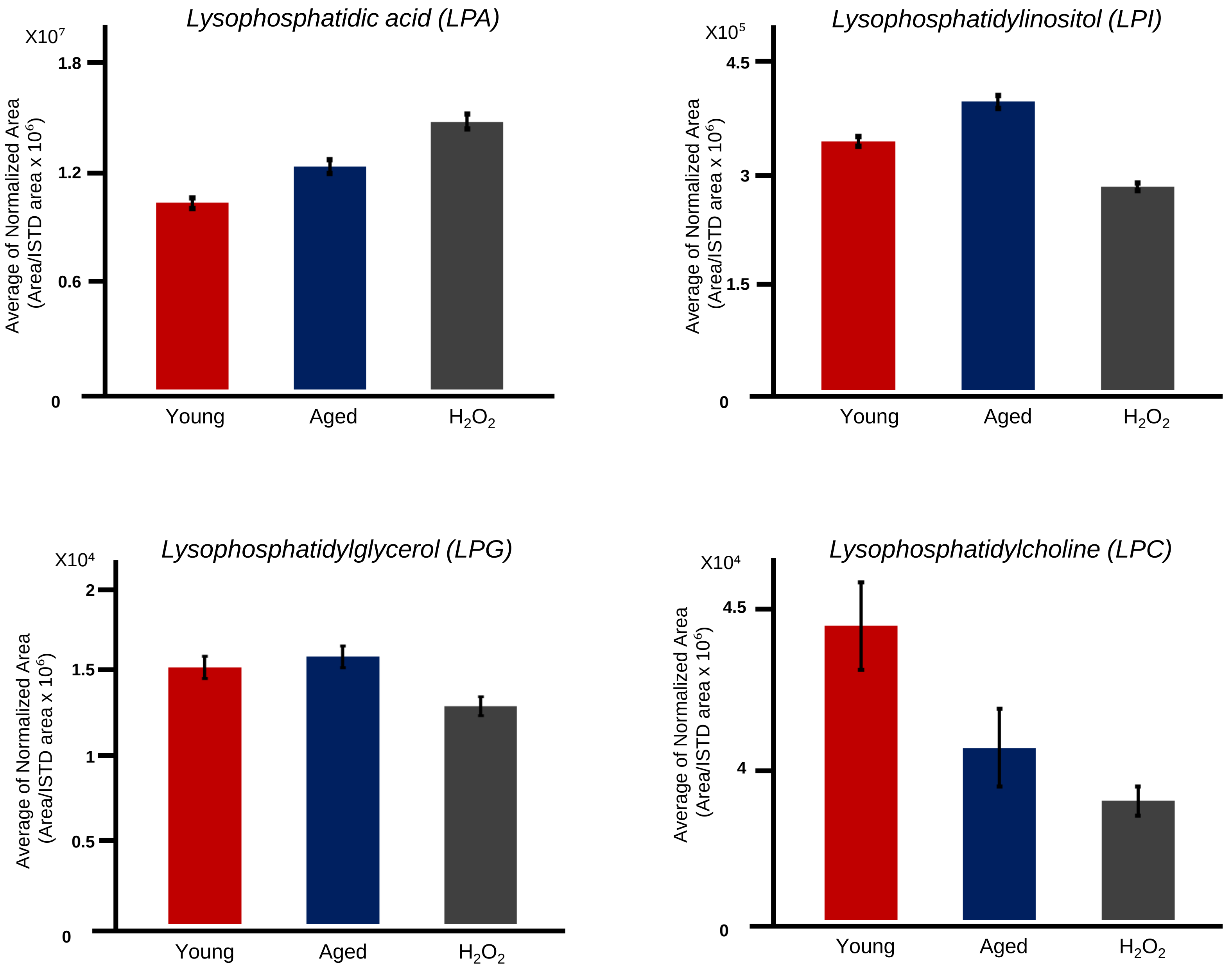

Lysophosphatidic acid (LPA)
X10^7
1.8
1.2
0.6
0
Average of Normalized Area (Area/ISTD area x 10^6)
Young
Aged
H2O2
Lysophosphatidylinositol (LPI)
X10^5
4.5
3
1.5
0
Average of Normalized Area (Area/ISTD area x 10^6)
Young
Aged
H2O2
Lysophosphatidylglycerol (LPG)
X10^4
2
1.5
1
0.5
0
Average of Normalized Area (Area/ISTD area x 10^6)
Young
Aged
H2O2
Lysophosphatidylcholine (LPC)
X10^4
4.5
4
0
Average of Normalized Area (Area/ISTD area x 10^6)
Young
Aged
H2O2

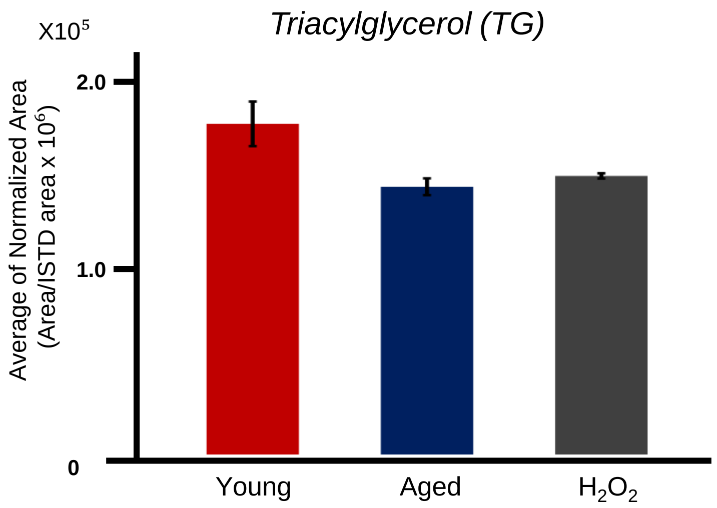

Triacylglycerol (TG)
X10^5
Average of Normalized Area
(Area/ISTD area x 10^6)
2.0
1.0
0
Young
Aged
H2O2
