# Supplementary material for: Age-Associated Lipidome Changes in Metaphase II Mouse Oocytes
Source: PLoS One. 2016 Feb 16;11(2):e0148577. doi: 10.1371/journal.pone.0148577 (PMC4755615; doi:10.1371/journal.pone.0148577)
Supplement: S1 Table — (PDF) [file pone.0148577.s002.pdf]

Table S1. Validation study of lipid analysis based on multiple reaction monitoring (MRM) and the limitation of detections (LODs) of each lipid standard (30 min).

Lipids[a)]	RT[b)] (min)	RSD (n=6) (%)[c)]		Correlation (R^2)	Linear range (pg)	LOD (pg)
		RT[d)]	Peak area[e)]			
TG	3.47	1.1	5.7	0.9992	0.1 – 2000	0.1
DG	1.46	0.6	5.4	0.9986	0.2 – 2000	0.2
ChE	13.47	0.8	5.9	0.9894	1000 – 50000	1000
Cholesterol	4.07	1.2	3.0	0.9664	1000-200000	1000
PC	1.66	1.1	4.9	0.9912	0.1 – 2000	0.1
PE	1.62	1.3	5.8	0.9954	0.2 – 2000	0.2
PG	1.46	0.8	8.9	0.9992	10 – 2000	10
LPC	1.36	1.0	4.8	0.9873	1 – 2000	1
LPE	1.38	1.2	5.7	0.9899	2 – 2000	2
LPG	1.27	0.9	3.5	0.9963	2 – 2000	2
SM	2.99	1.0	5.5	0.9956	1 – 2000	1
Cer	3.07	1.0	6.3	0.9962	1 – 2000	1
dCer	3.33	1.5	6.9	0.9984	1 – 2000	1
SO	1.34	0.9	3.4	0.9803	10 – 2000	10
SA	1.40	1.4	4.2	0.9984	10 – 2000	10
Methylated PS	1.88	0.2	4.2	0.9872	1 – 2000	1
Methylated PI	1.33	0.2	6.2	0.9954	100 – 50000	100
Methylated PA	2.14	0.3	4.8	0.9993	0.2 – 2000	0.2
Methylated LPS	1.57	0.2	5.4	0.9834	1 – 2000	1
Methylated LPI	1.32	0.2	6.5	0.9994	50 – 50000	50
Methylated LPA	2.04	0.8	7.3	0.9963	0.2 – 2000	0.2
Methylated Cer1P	3.75	0.2	4.8	0.9930	0.2 – 2000	0.2
Methylated So1P	1.39	0.1	4.5	0.9890	100 – 2000	100
Methylated Sa1P	1.44	0.2	4.4	0.9984	100 – 2000	100

[a)] Lipid standards used in this study were as follows: TG (11:1-11:1-11:1), DG (8:0-8:0), ChE (10:0), Cholesterol, PC (10:0-10:0), PE (10:0-10:0), PG (10:0-10:0), LPC (13:0), LPE (14:0), LPG (14:0), SM (d18:1-12:0), Cer (d18:1-12:0), dCer (d18:1-12:0), So (d17:1), Sa (d17:0), PS (10:0-10:0), PI (8:0-8:0), PA (10:0-10:0), LPS (17:1), LPI (13:0), LPA (14:0), Cer1P (d18:1-12:0), So1P (d17:1), and Sa1P (d17:0).

[b)] Retention time

[c)] Concentrations of each lipid were 0.1 mg/mL.

[d)] Relative retention time (compound/internal standard)

[e)] Relative peak area (compound/internal standard)
