# Supplementary material for: Age-Associated Lipidome Changes in Metaphase II Mouse Oocytes
Source: PLoS One. 2016 Feb 16;11(2):e0148577. doi: 10.1371/journal.pone.0148577 (PMC4755615; doi:10.1371/journal.pone.0148577)
Supplement: S2 Table — (PDF) [file pone.0148577.s003.pdf]

Table S2. Detected lipid species in oocytes.

Compound Name	Precursor Ion	Product Ion	Retention Time
Cer(d14:1-10:0)	398.4	208.1	1.757
Cer(d14:1-16:1)	480.4	208.1	1.877
Cer(d14:1-18:4)	502.4	208.1	1.995
Cer(d14:1-20:3)	532.4	208.1	1.971
Cer(d14:1-24:1)	592.4	208.1	2.159
Cer(d16:1-12:0)	454.4	236.1	3.808
Cer(d16:1-14:0)	482.4	236.1	4.903
Cer(d16:1-16:0)	510.4	236.1	6.317
Cer(d16:1-18:3)	532.4	236.1	1.919
Cer(d16:1-22:6)	582.4	236.1	3.848
Cer(d16:1-24:0)	622.4	236.1	15.153
Cer(d16:1-24:1)	620.4	236.1	13.533
Cer(d18:1-10:0)	454.4	264.1	3.821
Cer(d18:1-12:0)IS	482.4	264.1	4.853
Cer(d18:1-14:0)	510.4	264.1	6.32
Cer(d18:1-16:0)	538.4	264.1	8.327
Cer(d18:1-18:2)	562.4	264.1	4.02
Cer(d18:1-20:5)	584.4	264.1	4.017
Cer(d18:1-24:0)	650.4	264.1	17.18
Cer(d18:1-24:1)	648.4	264.1	14.993
Cer1P(d14:1-10:0)	506.5	208.1	1.778
Cer1P(d14:1-16:0)	590.5	208.1	2.073
Cer1P(d14:1-20:4)	638.5	208.1	1.633
Cer1P(d14:1-24:1)	700.5	208.1	11.684
Cer1P(d14:1-8:0)	478.5	208.1	1.5
Cer1P(d16:1-12:0)	562.5	236.1	3.722
Cer1P(d16:1-14:0)	590.5	236.1	4.728
Cer1P(d18:1-10:0)	562.5	264.1	3.74
Cer1P(d18:1-12:0)IS*	590.5	264.1	4.749
Cer1P(d18:1-14:0)	618.5	264.1	6.191
Cer1P(d18:1-16:0)	646.5	264.1	8.115
Cer1P(d18:1-16:1)	644.5	264.1	6.19
Cer1P(d18:1-18:0)	674.5	264.1	10.255
Cer1P(d18:1-20:5)	692.5	264.1	4.769
ChE(10:0)IS	558.5	369.2	18.14
ChE(18:0)	670.6	369.2	21.453
ChE(18:1)	668.6	369.2	20.701

ChE(18:2)	666.6	369.2	19.927
ChE(18:4)	662.6	367.2	17.18
ChE(20:3)	692.6	369.2	20.759
ChE(20:4)	690.6	369.2	20.148
ChE(20:5)	688.6	369.2	19.294
ChE(22:0)	726.7	369.2	23.168
ChE(22:1)	724.6	369.2	22.699
ChE(22:6)	714.6	369.2	19.516
ChE(24:0)	754.7	369.2	23.631
ChE(24:1)	752.7	369.2	23.284
Cholesterol	404	369.2	6.125
dCer(d14:0-18:4)	504.4	210.1	2.006
dCer(d18:0-12:0)IS	484.4	266.1	5.337
dCer(d18:0-16:0)	540.4	266.1	9.083
dCer(d18:0-18:2)	564.4	266.1	4.064
dCer1P(d14:1-16:0)	592.5	210.1	2.415
dCer1P(d14:1-20:0)	648.5	210.1	8.82
dCer1P(d14:1-20:5)	638.5	210.1	1.654
dCer1P(d14:1-8:0)	480.5	210.1	1.484
dCer1P(d16:1-12:0)	564.5	238.1	2.253
dCer1P(d16:1-14:0)	592.5	238.1	4.732
dCer1P(d16:1-16:1)	618.5	238.1	2.302
dCer1P(d16:1-18:0)	648.5	238.1	8.853
dCer1P(d16:1-18:3)	642.5	238.1	6.707
dCer1P(d16:1-20:3)	670.5	238.1	8.845
dCer1P(d18:1-12:0)	592.5	266.1	4.734
dCer1P(d18:1-16:0)IS	648.5	266.1	8.845
dCer1P(d18:1-18:3)	670.5	266.1	8.876
DG(16:0)IS	362.2	327.2	2.123
DG(28:2)	526.4	491.4	2.864
DG(28:4)	522.4	487.4	1.952
DG(28:5)	520.3	485.3	1.482
DG(30:0)	558.5	523.5	9.299
DG(30:1)	556.4	521.4	7.553
DG(32:0)	586.5	551.5	11.482
DG(32:1)	584.5	549.5	9.62
DG(32:2)	582.5	547.5	7.948
DG(34:0)	614.5	579.5	13.705
DG(34:1)	612.5	577.5	11.638

DG(34:2)	610.5	575.5	9.655
DG(34:3)	608.5	573.5	8.002
DG(34:5)	604.4	569.4	5.743
DG(34:6)	602.4	567.4	4.863
DG(36:0)	642.6	607.6	15.821
DG(36:3)	636.5	601.5	9.819
DG(36:4)	634.5	599.5	7.931
DG(36:5)	632.5	597.5	6.386
DG(36:7)	628.4	593.4	2.5
DG(36:8)	626.4	591.4	1.567
DG(42:11)	704.5	669.5	2.98
LPA(10:0)	355.3	229.2	1.722
LPA(14:0)	411.3	285.2	1.943
LPA(16:0)	439.3	313.2	2.484
LPA(16:1)	437.3	311.2	1.79
LPA(17:0)IS	453.3	327.2	2.705
LPA(18:0)	467.3	341.2	3.004
LPA(18:1)	465.3	339.2	2.538
LPA(18:2)	463.3	337.2	2.206
LPA(18:3)	461.3	335.2	1.445
LPA(18:4)	459.3	333.2	1.382
LPA(20:5)	485.3	359.2	1.984
LPA(8:0)	327.3	201.2	1.61
LPC(10:0)	412.2	183.9	1.617
LPC(13:0)IS	454.1	183.9	1.795
LPC(14:0)	468.1	183.9	1.922
LPC(16:0)	496.1	183.9	2.142
LPC(16:1)	494.1	183.9	1.905
LPC(18:0)	524.1	183.9	2.528
LPC(18:1)	522.1	183.9	2.165
LPC(18:2)	520.1	183.9	1.926
LPC(18:3)	518.1	183.9	1.822
LPC(18:4)	516.1	183.9	1.736
LPC(20:0)	552.1	183.9	3.042
LPC(20:1)	550.1	183.9	2.593
LPC(20:3)	546.1	183.9	1.982
LPC(20:4)	544.1	183.9	1.854
LPC(20:5)	542.1	183.9	1.712
LPC(22:6)	568.1	183.9	2.28

LPC(24:0)	608.1	183.9	4.929
LPC(24:1)	606.1	183.9	4.648
LPE(14:0)IS	426.3	285.2	1.931
LPE(18:1)	480.3	339.2	2.191
LPE(18:2)	478.3	337.2	1.935
LPE(18:3)	476.3	335.2	1.846
LPE(20:1)	508.3	367.2	2.148
LPE(20:5)	500.3	359.2	1.973
LPE(22:0)	538.3	397.2	2.532
LPE(22:4)	530.3	389.2	2.084
LPG(14:0)IS	457.1	285.2	1.813
LPG(16:0)	485.1	313.2	2.058
LPG(16:1)	483.1	311.2	1.86
LPG(18:1)	511.1	339.2	1.967
LPG(18:4)	505.1	333.2	1.857
LPG(22:1)	567.1	395.2	2.401
LPI(13:0)IS	545.2	271.1	1.791
LPI(14:0)	559.2	285.1	1.859
LPI(18:0)	615.2	341.1	3.271
LPI(18:3)	609.2	335.1	1.474
LPI(8:0)	475.2	201.1	1.503
LPS(10:0)	442.3	229.2	1.624
LPS(16:0)	526.3	313.2	1.628
LPS(17:1)IS	538.3	325.2	2.051
LPS(18:0)	554.3	341.2	2.769
LPS(18:1)	552.3	339.2	2.293
LPS(18:2)	550.3	337.2	1.959
LPS(18:3)	548.3	335.2	1.485
LPS(20:4)	574.3	361.2	2.247
LPS(22:6)	598.3	385.2	2.312
PA(18:0)	481.5	355.3	2.377
PA(20:0)IS	509.5	383.3	2.801
PA(26:1)	591.5	465.3	4.744
PA(28:1)	619.5	493.3	6.825
PA(28:4)	613.5	487.3	4.741
PA(28:5)	611.5	485.3	1.431
PA(30:0)	649.5	523.3	8.871
PA(30:1)	647.5	521.3	7.42
PA(30:3)	643.5	517.3	5.962

PA(32:3)	671.5	545.3	8.871
PC(18:0)	538.3	183.9	1.996
PC(20:0)IS	566.3	183.9	2.303
PC(22:0)	594.4	183.9	2.761
PC(24:0)	622.4	183.9	3.4
PC(24:1)	620.4	183.9	2.194
PC(26:0)	650.4	183.9	4.424
PC(26:4)	642.4	183.9	1.68
PC(28:0)	678.5	183.9	5.51
PC(30:0)	706.5	183.9	8.524
PC(30:1)	704.5	183.9	7.603
PC(30:2)	702.5	183.9	5.768
PC(30:6)	694.5	183.9	2.57
PC(32:0)	734.5	183.9	9.261
PC(32:1)	732.5	183.9	7.411
PC(32:2)	730.5	183.9	5.705
PC(32:3)	728.5	183.9	4.627
PC(34:0)	762.5	183.9	11.515
PC(34:1)	760.5	183.9	9.364
PC(34:2)	758.5	183.9	7.481
PC(34:3)	756.5	183.9	5.857
PC(34:4)	754.5	183.9	5.16
PC(34:5)	752.5	183.9	4.268
PC(36:0)	790.5	183.9	13.604
PC(36:1)	788.5	183.9	11.516
PC(36:2)	786.5	183.9	9.459
PC(36:3)	784.5	183.9	7.569
PC(36:4)	782.5	183.9	6.804
PC(36:5)	780.5	183.9	5.313
PC(36:6)	778.5	183.9	4.661
PC(36:7)	776.5	183.9	3.452
PC(38:0)	818.5	183.9	16.252
PC(38:1)	816.5	183.9	14.098
PC(38:2)	814.5	183.9	9.559
PC(38:3)	812.5	183.9	8.832
PC(38:4)	810.5	183.9	6.907
PC(38:5)	808.5	183.9	6.075
PC(38:6)	806.5	183.9	4.696
PC(40:1)	844.5	183.9	15.788

PC(40:2)	842.5	183.9	13.907
PC(40:3)	840.5	183.9	11.652
PC(40:4)	838.5	183.9	10.381
PC(40:5)	836.5	183.9	8.321
PC(40:6)	834.5	183.9	7.949
PC(40:7)	832.5	183.9	6.131
PC(40:8)	830.5	183.9	4.917
PC(42:1)	872.5	183.9	17.995
PC(42:10)	854.5	183.9	5.369
PC(42:11)	852.5	183.9	4.465
PC(42:2)	870.5	183.9	15.615
PC(42:5)	864.5	183.9	10.196
PC(42:6)	862.5	183.9	8.391
PC(42:7)	860.5	183.9	7.509
PC(42:8)	858.5	183.9	6.012
PC(42:9)	856.5	183.9	4.394
PE(16:0)	468.2	327.3	1.8
PE(18:0)	496.3	355.3	1.987
PE(20:0)IS	524.4	383.3	2.293
PE(22:0)	552.4	411.3	2.723
PE(24:0)	580.4	439.3	3.381
PE(24:1)	578.4	437.3	2.228
PE(32:0)	692.4	551.3	9.133
PE(32:1)	690.4	549.3	7.321
PE(34:0)	720.4	579.3	11.492
PE(34:1)	718.4	577.3	9.261
PE(34:2)	716.4	575.3	7.415
PE(34:3)	714.4	573.3	5.687
PE(36:1)	746.4	605.3	11.382
PE(36:2)	744.4	603.3	9.403
PE(36:3)	742.4	601.3	7.554
PE(36:4)	740.4	599.3	6.848
PE(38:4)	768.4	627.3	8.809
PE(38:5)	766.4	625.3	6.917
PE(44:5)	850.4	709.3	11.334
PG(20:0)IS	572.4	383.3	2.053
PG(34:0)	768.4	579.3	9.719
PG(34:1)	766.4	577.3	7.635
PG(34:2)	764.4	575.3	6.067

PG(36:1)	794.4	605.3	9.707
PG(36:2)	792.4	603.3	7.904
PG(36:3)	790.4	601.3	6.181
PG(36:4)	788.4	599.3	5.587
PG(38:2)	820.4	631.3	9.857
PG(38:4)	816.4	627.3	7.309
PG(38:5)	814.4	625.3	5.663
PI(16:0)IS	601.6	327.3	3
PI(20:0)	657.6	383.3	2.139
PI(22:0)	685.6	411.3	2.653
PI(32:0)	825.6	551.3	21.514
PI(32:1)	823.6	549.3	21.083
PI(32:2)	821.6	547.3	20.161
PI(34:0)	853.6	579.3	22.062
PI(34:1)	851.6	577.3	21.459
PI(34:2)	849.6	575.3	20.2
PI(36:0)	881.6	607.3	22.525
PI(36:2)	877.6	603.3	21.473
PS(20:0)IS	596.6	383.3	2.3
PS(22:0)	624.6	411.3	2.771
PS(26:1)	678.6	465.3	4.718
PS(26:4)	672.6	459.3	2.368
PS(28:0)	708.6	495.3	7.401
PS(30:0)	736.6	523.3	8.81
PS(32:6)	752.6	539.3	2.191
PS(36:1)	818.6	605.3	11.341
PS(36:2)	816.6	603.3	9.568
PS(36:3)	814.6	601.3	8.895
SA1P(d14:0)	354.3	210.1	1.499
SA1P(d17:0)IS	396.3	252.1	1.94
SM(d18:1-10:0)	619.3	183.9	3.417
SM(d18:1-12:0)IS	647.3	183.9	4.366
SM(d18:1-14:0)	675.3	183.9	5.656
SM(d18:1-16:0)	703.3	183.9	7.537
SM(d18:1-16:1)	701.3	183.9	5.712
SM(d18:1-18:0)	731.3	183.9	9.602
SM(d18:1-18:1)	729.3	183.9	7.538
SM(d18:1-22:0)	787.3	183.9	9.364
SM(d18:1-22:1)	785.3	183.9	7.494

SM(d18:1-24:0)	815.3	183.9	16.247
SM(d18:1-24:1)	813.3	183.9	14.064
SO(d14:1)	244.3	208.1	1.51
SO(d17:1)IS	286.3	250.1	1.871
SO1P(d14:1)	352.3	208.1	1451
SO1P(d17:1)IS	394.3	250.1	1.877
TG(33:3)IS	608.5	407.3	5.36
TG(42:0)	740.68	495.5	18.986
TG(42:1)	738.66	521.5	17.753
TG(42:3)	734.63	545.5	14.422
TG(44:0)	768.71	523.5	19.9
TG(44:1)	766.69	521.5	19.128
TG(46:0)	796.74	551.5	20.647
TG(46:1)	794.72	549.5	19.984
TG(46:2)	792.71	547.5	19.123
TG(48:0)	824.77	607.5 or 551.5	19.278 or 21.254
TG(48:1)	822.76	549.5	20.67
TG(48:2)	820.74	547.5	19.962
TG(48:3)	818.72	547.5	19.351
TG(50:0)	852.8	579.5	22.033
TG(50:1)	850.8	577.5	21.478
TG(50:2)	848.77	575.5	20.888
TG(50:3)	846.76	575.5	20.409
TG(50:4)	844.74	573.5	19.588
TG(52:0)	880.82	607.5	22.471
TG(52:1)	878.82	605.5	21.963
TG(52:2)	876.8	603.5	21.44
TG(52:3)	874.79	601.5	20.874
TG(52:4)	872.77	599.5	20.251
TG(52:5)	870.76	597.5	19.516
TG(54:0)	908.82	607.5	22.863
TG(54:1)	906.82	607.5	22.442
TG(54:10)	888.76	595.5	16.093
TG(54:2)**	904.82	603.5	22 or 21.426
TG(54:3)**	902.82	603.5	21.431 or 20.861
TG(54:4)	900.82	603.5	20.853
TG(54:5)	898.79	601.5	20.221
TG(54:6)	896.77	599.5	19.415
TG(54:7)	894.76	597.5	18.57

TG(54:8)	892.76	599.5	17.383
TG(56:0)	936.82	551.5	23.188
TG(56:1)**	934.82	633.5 or 549.5	22.39 or 22.909
TG(56:3)	930.82	631.5	21.948
TG(56:4)	928.82	631.5	21.421
TG(56:5)	926.82	603.5	20.946
TG(56:6)	924.8	625.5	20.594
TG(56:7)	922.79	577.5	20.152
TG(58:0)	964.82	579.5	23.461
TG(58:1)	962.82	577.5	23.228
TG(58:10)	944.82	623.5	18.59
TG(58:3)	958.82	603.5	22.331
TG(58:4)	956.82	601.5	21.834
TG(58:5)	954.82	635.5	21.47
TG(60:1)	990.82	605.5	23.552
TG(60:10)	972.82	627.5	19.55
TG(60:11)	970.82	625.5	18.933
TG(60:3)	986.82	603.5	22.759
TG(60:4)	984.82	599.5	22.731
TG(60:5)	982.82	663.5	21.888

* IS: Internal Standard

** Regioisomer
