# Supplementary material for: Age-Associated Lipidome Changes in Metaphase II Mouse Oocytes
Source: PLoS One. 2016 Feb 16;11(2):e0148577. doi: 10.1371/journal.pone.0148577 (PMC4755615; doi:10.1371/journal.pone.0148577)
Supplement: S3 Table — Data from three technical replicates are shown. (PDF) [file pone.0148577.s004.pdf]

Table S3. Lipid species uniquely expressed in each comparison. Data from three technical replicates are shown.

Unique lipidome in aged oocytes

Assignment	*Young oocytes*			*Aged oocytes*		
	**R1***	**R2**	**R3**	**R1**	**R2**	**R3**
TG(60:11)	N.Q	N.Q	N.Q	655	702	844
TG(60:10)	539	N.Q	N.Q	984	1215	1274
TG(60:5)	N.Q	N.Q	N.Q	789	703	641
PG(36:4)	N.Q	N.Q	603.00	692	1050	1145
PC(42:8)	N.Q	N.Q	N.Q	713	1001	800
PC(42:9)	N.Q	N.Q	N.Q	544	636	N.Q
dCer(d14:0-18:4)	N.Q	533	N.Q	652	996	1004.0
DG(28:2)	N.Q	N.Q	N.Q	N.Q	759	597
DG(34:3)	N.Q	N.Q	666.00	805	516	745
LPC(20:1)	N.Q	N.Q	N.Q	664	N.Q	808
LPG(22:1)	N.Q	N.Q	532.00	506	567	666.0
LPC(14:0)	714	N.Q	0.00	700	653	684

Unique lipidome in young oocytes

Assignment	*Young oocytes*			*Aged oocytes*		
	**R1**	**R2**	**R3**	**R1**	**R2**	**R3**
PC(34:4)	720	763	664.00	N.Q	N.Q	N.Q
PE(32:0)	769	641	N.Q	N.Q	584	N.Q
TG(58:4)	1008	1042	1251.00	884	N.Q	N.Q

* R : Technical replication

** N.Q : Not quantifiable

Unique lipidome in H_2O_2-treated young oocytes						
Assignment	***Young oocytes***			***H_2O_2-treated***		
	**R1***	**R2**	**R3**	**R1**	**R2**	**R3**
TG(60:5)	N.Q	N.Q	N.Q	510	740	646
PG(36:4)	N.Q	N.Q	603	N.Q	592	628
LPG(22:1)	N.Q	N.Q	532	576	635	608
LPE(20:5)	N.Q	N.Q	N.Q	538	608	577
LPC(14:0)	714	N.Q	N.Q	614	533	757
DG(34:3)	N.Q	N.Q	666	631	677	N.Q
DG(28:2)	N.Q	N.Q	N.Q	960	975	N.Q

Unique lipidome in young oocytes						
Assignment	***Young mice***			***H_2O_2-treated***		
	**R1**	**R2**	**R3**	**R1**	**R2**	**R3**
ChE(18:0)	508	640	511	N.Q	N.Q	N.Q
ChE(20:5)	625	590	N.Q	N.Q	N.Q	N.Q
LPC(20:3)	N.Q	550	1115	N.Q	N.Q	N.Q
PC(30:6)	1656	1418	1277	N.Q	N.Q	N.Q
PC(34:4)	720	763	664	N.Q	N.Q	N.Q
PC(38:1)	626	N.Q	902	N.Q	N.Q	N.Q
PG(34:0)	513	588	N.Q	N.Q	N.Q	808
PG(38:5)	591	676	N.Q	563	N.Q	N.Q
PI(32:2)	N.Q	553	535	N.Q	894	N.Q

* R : Technical replication

** N.Q : Not quantifiable
